# Sphenopalatine ganglion stimulation: a comprehensive evaluation across diseases in randomized controlled trials

**DOI:** 10.3389/fneur.2024.1352145

**Published:** 2024-05-15

**Authors:** Lingli Qin, Dian Chen, Xian Li, Yue Gao, Wanying Xia, Hanxi Dai, Linjie Qiu, Jinsheng Yang, Lu Zhang

**Affiliations:** ^1^Xiyuan Hospital, China Academy of Chinese Medical Sciences, Beijing, China; ^2^Graduate School, Beijing University of Chinese Medicine, Beijing, China; ^3^Institute of Basic Theory for Chinese Medicine, China Academy of Chinese Medical Sciences, Beijing, China

**Keywords:** sphenopalatine ganglion stimulation, allergic rhinitis, ischemic strokes, cluster headache, primary trigeminal neuralgia, chronic tension-type headache, refractory facial paralysis, pediatric chronic secretory otitis

## Abstract

**Background:**

Current literature extensively covers the use of sphenopalatine ganglion stimulation (SPGs) in treating a broad spectrum of medical conditions, such as allergic rhinitis, cluster headaches, and strokes. Nevertheless, a discernible gap in the systematic organization and analysis of these studies is evident. This paper aims to bridge this gap by conducting a comprehensive review and analysis of existing literature on SPGs across various medical conditions.

**Methods:**

This study meticulously constructed a comprehensive database through systematic computerized searches conducted on PubMed, Embase, CNKI, Wanfang, VIP, and CBM up to May 2022. The inclusion criteria encompassed randomized controlled trials (RCTs) published in either Chinese or English, focusing on the therapeutic applications of SPGs for various medical conditions. Both qualitative and quantitative outcome indicators were considered eligible for inclusion.

**Results:**

This comprehensive study reviewed 36 publications, comprising 10 high-quality, 23 medium-quality, and three low-quality articles. The study investigated various diseases, including allergic rhinitis (AR), ischemic strokes (IS), cluster headache (CH), primary trigeminal neuralgia (PTN), pediatric chronic secretory otitis (PCSO), refractory facial paralysis (RFP), chronic tension-type headache (CTTH), as well as the analysis of low-frequency sphenopalatine ganglion stimulation (LF-SPGs) in chronic cluster headache (CCH) and the impact of SPGs on Normal nasal cavity function (NNCF). SPGs demonstrate efficacy in the treatment of AR. Regarding the improvement of rhinoconjunctivitis quality of life questionnaire (RQLQ) scores, SPGs are considered the optimal intervention according to the SUCRA ranking. Concerning the improvement in Total Nasal Symptom Score (TNSS), Conventional Acupuncture Combined with Tradiational Chinese Medicine (CA-TCM) holds a significant advantage in the SUCRA ranking and is deemed the best intervention. In terms of increasing Effective Rate (ER), SPGs outperformed both conventional acupuncture (CA) and Western Medicine (WM; *P* < 0.05). In the context of SPGs treatment for IS, the results indicate a significant improvement in the 3-month outcomes, as evaluated by the modified Rankin Scale (mRS) in the context of Cerebral Cortical Infarction (CCI; *P* < 0.05). In the treatment of CH with SPGs, the treatment has been shown to have a statistically significant effect on the relief and disappearance of headaches (*P* < 0.05). The impact of SPGs on NNCF reveals statistically significant improvements (*P* < 0.05) in nasal airway resistance (NAR), nasal cavity volume (NCV), exhaled nitric oxide (eNO), substance P (SP), vasoactive intestinal peptide (VIP) and neuropeptide Y (NPY). SPGs treatments for PCSO, RFP, and CTTH, when compared to control groups, yielded statistically significant results (*P* < 0.05).

**Conclusion:**

SPGs demonstrate significant effectiveness in the treatment of AR, IS, and CH. Effective management of CCH may require addressing both autonomic dysregulation and deeper neural pathways. However, additional high-quality research is essential to clarify its effects on NNCF, PTN, PCSO, RFP, and CTTH.

**Systematic Review Registration:**

PROSPERO, identifier CRD42021252073, https://www.crd.york.ac.uk/PROSPERO/display_record.php?RecordID=312429.

## 1 Introduction

The sphenopalatine ganglion (SPG) is a significant extracranial parasympathetic ganglion, comprising both autonomic and sensory nerves. It is situated beneath the maxillary nerve in the pterygopalatine fossa (PPF) (1). Preganglionic parasympathetic fibers travel from the superior salivary nucleus through the facial nerve to the geniculate ganglion. Here, they divide into the greater petrosal nerve, which merges with the deep petrosal nerve to form the Vidian nerve (2). postganglionic sympathetic fibers, originating from the superior cervical ganglion, traverse the internal cervical plexus to create the deep Petrosal nerve. This nerve joins the greater petrosal nerve, forming the Vidian nerve as it crosses the SPG (3). Sensory fibers have their origin in the maxillary nerve branches and pass through the SPG (4).

Postganglionic parasympathetic fibers, arising from the SPG, extend to various regions, including the nasal cavity, palate, nasopharynx, and oropharynx, through the ophthalmic and maxillary branches of the trigeminal nerve. Additionally, some postganglionic parasympathetic nerve branches have been observed to course medially and superiorly from the SPG, penetrating the orbital cavity and providing parasympathetic innervation to the meninges and cerebral vessels (5).

The discovery of the SPG as the initial relay station for autonomic fibers following their emergence from the pons suggests its potential therapeutic applications in cases of autonomic imbalance. Additionally, SPG may serve a vital role as a vasodilator in protecting the brain from ischemic events such as strokes (6). SPG has shown promise in improving neurological outcomes by reducing the semidark band, shrinking cerebral infarction size, enhancing neuronal survival, and maintaining the integrity of the blood-brain barrier (7, 8). In animal studies involving healthy rats (9), cats (10), dogs (11), and primates (12), SPGs has demonstrated its ability to widen the ipsilateral anterior circumflex intradural artery of Willis and reduce infarction severity following permanent middle cerebral artery occlusion in rats (13, 14). Clinical trials in humans have indicated the safety of SPG intervention for patients 8–24 h after acute IS who are not eligible for thrombolytic therapy (15).

Additionally, crucial SPG nerve fibers, transmitting signals from the trigeminal nerve, play pivotal roles in various pain syndromes, encompassing atypical facial discomfort, trigeminal autonomic cephalalgia (TAC), and pain resulting from herpes infections. TAC is particularly notable for its intracranial autonomic characteristics. This arises due to the activation of the trigeminal-autonomic reflex when the trigeminal afferent nerve stimulates the superior salivary nucleus, leading to the release of vasoactive peptides such as acetylcholine (AC), vasoactive intestinal peptide (VIP), and nitric oxide (NO). These peptides induce plasma protein (PP) extravasation and neurogenic inflammation (16, 17). High-frequency electrical SPGs effectively alleviates acute pain and proactively reduces the frequency of headache attacks by suppressing parasympathetic output through transmitter synthesis and release depletion (18).

Notably, the SPG stands as the singular ganglion accessible to the external environment through the nasal mucosa. Numerous trials have demonstrated the effectiveness of SPGs in treating rhinitis. The mechanism underlying SPG's efficacy in rhinitis therapy involves the enhancement of neurological, endocrine, and immune system control. This is achieved by down-regulating pro-inflammatory neuropeptides, neurotrophins, Th2 cytokines, and pro-inflammatory cytokines, thereby shifting the Th1/Th2 balance toward Th1 (19–21). The SPG has garnered substantial attention in the literature for its potential therapeutic applications, ranging from case reports to pilot studies and experimental investigations. This article adds to the existing body of knowledge by presenting findings from randomized controlled pilot trials, providing valuable insights into its therapeutic potential. The study aimed to accomplish the following objectives:

- Review and analysis: we will undertake a systematic review and analysis of SPGs to deeply explore its therapeutic range and varied applications in treating multiple diseases. This endeavor aims to develop an overarching conceptual framework for its treatment modalities.
- Meta-analysis and network meta-analysis: through meta-analysis and network meta-analysis, we intend to amalgamate and critically assess the corpus of existing research, thereby enriching our comprehension of SPGs' therapeutic impact across a spectrum of diseases. This effort will encompass a quantitative evaluation of study heterogeneity and the appraisal of SPGs' relative effectiveness, utilizing both direct and indirect evidence.
- Comprehensive analysis and future outlook: we will perform a detailed analysis to delineate and encapsulate the therapeutic merits and strengths of SPGs in ameliorating diseases. Furthermore, recognizing the constraints of existing studies and the variability in treatment outcomes, we will delineate directions for future research, with a particular focus on elucidating treatment mechanisms, refining efficacy assessments, and exploring long-term effects.

## 2 Data and methods

### 2.1 Inclusion criteria

- Population: inclusive of patients diagnosed with various diseases.
- Intervention: utilization of sphenopalatine ganglion stimulation (SPGs).
- Comparison: involving patients who do not receive any form of treatment, healthy controls, and individuals undergoing alternative therapeutic approaches.
- Outcomes: assessment of the reduction in illness severity, either qualitatively or quantitatively.
- Setting: inclusion criteria are limited to RCTs published in both Chinese and English, with a specific focus on studies conducted in both China and internationally.

### 2.2 Literature retrieval

An extensive search for relevant articles was conducted using multiple databases, including PubMed, Embase, CNKI, Wanfang VIP, and the Chinese Biomedical Literature Database (CBM). To ensure comprehensiveness, we employed a combination of keywords and free-text searches, customizing our approach to meet the specific requirements of each database. Search terms included “sphenopalatine ganglion,” “pterygopalatine ganglion,” “Xinwu acupoint,” “Treat the third nasal acupoint,” “neurostimulation,” “acupuncture,” and “stimulation.” Furthermore, we utilized published systematic reviews to identify relevant clinical studies, reducing the risk of overlooking important research. Our database search covered the period from the inception of the databases to May 2022.

### 2.3 Selection process and data extraction

Two researchers independently screened the retrieved articles based on predefined inclusion criteria and extracted relevant data. Any discrepancies in their assessments were resolved through discussion and consensus. Data extraction was performed using a customized data extraction table, which encompassed details such as author, sample size, quality, interventions, outcomes, course, dropouts, adverse reactions, and follow-up.

### 2.4 Risk of bias and quality of evidence evaluation

The risk of bias (ROB) assessment, as recommended by the Cochrane Institute (22) was employed. A comprehensive evaluation of the included articles for ROB was conducted by two researchers, with any disagreements being amicably resolved through discussion. The ROB assessment considered several key factors, including the randomization method, blinding of both participants and researchers, blinding of evaluators, allocation concealment, completeness of outcomes, selective result reporting, and identification of potential sources of bias. Each RCT included in the study was categorized as having a low, high, or unclear ROB. Subsequently, the included trials were categorized as high, low, or moderate quality based on the stipulated criteria.

- High quality: the entries for randomization method and allocation concealment were both found to have a low ROB, while all other items had an unclear bias or low ROB.
- Low quality: regardless of the risk of the other item, if any of the two entries for randomization method and allocation concealment was rated as having a high ROB.
- Moderate quality: both the randomization method and allocation concealment were rated as having a low ROB, whereas the remaining five entries were rated as having a high ROB.

### 2.5 Planned methods of analysis

#### 2.5.1 Meta analysis

Statistical analysis was conducted using RevMan 5.3, where the mean difference (MD) and its 95% confidence interval (CI) were expressed for continuous variables. The extent of heterogeneity amongst the included studies was assessed quantitatively using the chi-square test (test level = 0.1) paired with *I*^2^. A fixed-effects model was utilized where there was no statistical heterogeneity (*P* > 0.05 or *I*^2^ < 50%) between the study results. If there was statistical heterogeneity between the study results (*P* < 0.05 or *I*^2^ > 50%), the sources of heterogeneity were investigated further, and a meta-analysis was performed after eliminating the effect of significant clinical heterogeneity. Subgroup analysis or sensitivity analysis were used to address significant clinical heterogeneity, or just descriptive analysis was undertaken. When appropriate, funnel plots are supplied to detect publication bias and small sample effects.

#### 2.5.2 Network meta analysis

A random effects model was utilized for direct comparison of two pairs, followed by a network meta-analysis. Stata 13.0 Mesh Meta-analysis with Network Coding. The surface under the cumulative ranking (SUCRA) curve was used as evaluation indicators to rank the therapeutic benefits of the therapies for comparison. According to the global inconsistency test, *P* < 0.05 shows that the inconsistency is significant and cannot be explained by the consistency model. The nodal split approach was used to analyze the model's local inconsistency, and *P* < 0.05 indicated the presence of local inconsistency. Correcting funnel plots enables the detection of small sample utility or publication bias.

## 3 Results

### 3.1 Overall summary

**Figure 1:**
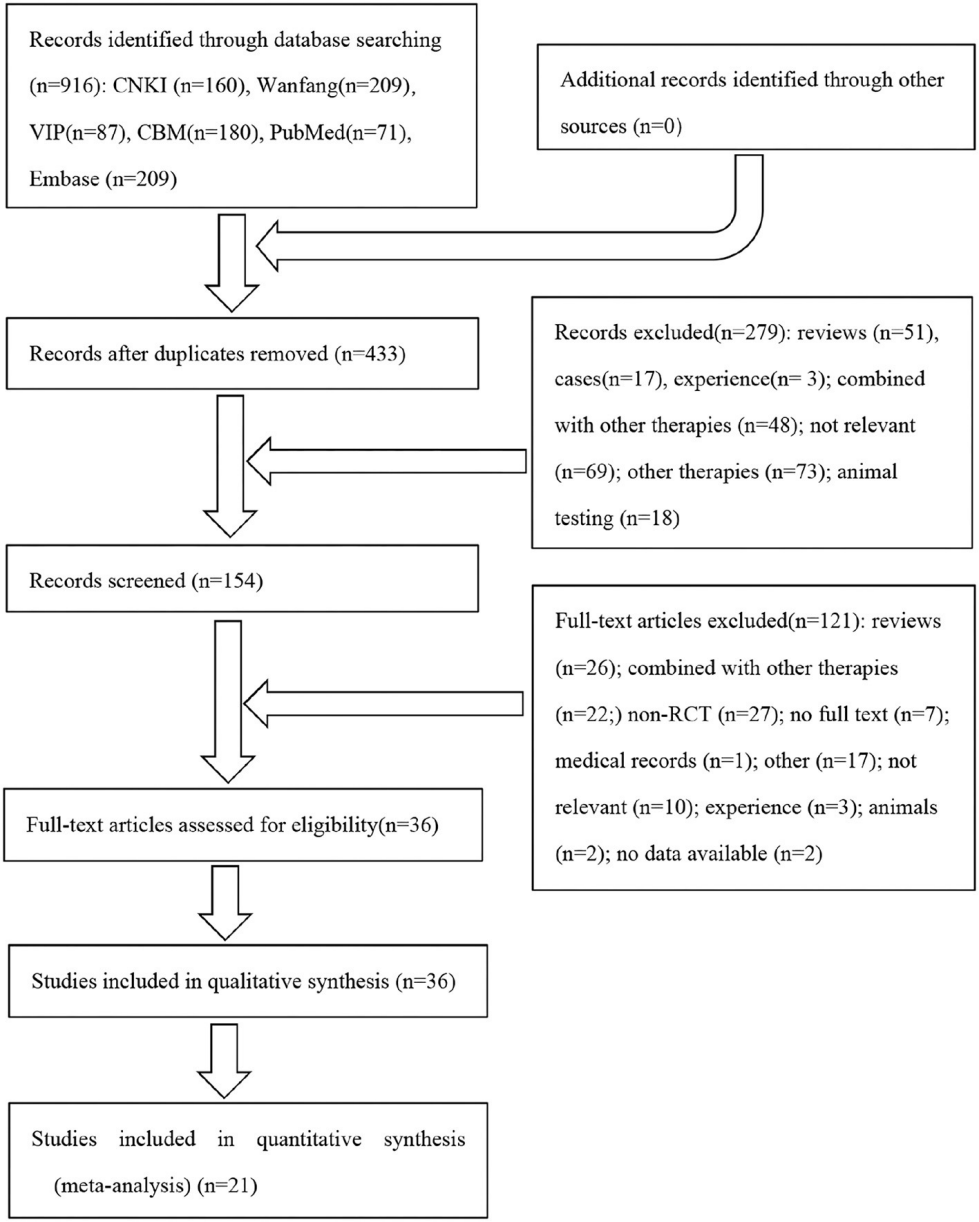
Flowchart of the study selection process.

A total of 916 articles were initially identified: 71 from PubMed, 209 from Embase, 160 from CNKI, 209 from Wanfang, 87 from VIP, and 180 from CBM. We conducted a thorough removal of duplicate articles using Endnote X9, resulting in 433 unique articles for subsequent systematic screening. Ultimately, 36 articles met the inclusion criteria. For a detailed visual representation of the literature retrieval process, please refer to Figure 1.

The study incorporated a comprehensive total of 36 RCTs, with 21 focusing on AR (23–43), two on IS (44, 45), two on CH (46, 47), three on PTN (48–50), three on NNCF (51–53), one on pediatric chronic secretory otitis (PCSO) (54), one on refractory facial paralysis (RFP) (55), one on chronic tension-type headache (CTTH) (56), and two on the analysis of LF-SPGs in CCH (57, 58). The summarizes the characteristics is shown in Table 1.

**Table 1:** Characteristics of the included studies.

**Disease**	**References**	**Sample size**	**Quality**	**Interventions**	**Outcomes**	**Course**	**Dropouts/adverse reactions**	**Follow-up**
AR	Shen (23)	30	Moderate	SPGs	SSS, RQLQ, ER	4 weeks	Neither group exhibited any abnormal adverse reactions associated with the treatment, and there were no instances of dropout. Within the SPGs group, two patients encountered local bleeding at the acupuncture site; however, this was promptly managed with compression, enabling the continuation of their treatment. Additionally, one patient in the SPGs group had a needle fainting reaction following needle removal, which resolved after a period of rest. The WM group reported no adverse drug reactions	3 months
		30		WM				
AR	Dong (24)	31	Moderate	SPGs	SSS, RQLQ	4 weeks	Not mentioned	Not mentioned
		31		WM				
AR	Song (25)	32	Moderate	SPGs	ER, IgE	4 weeks	Not mentioned	Not mentioned
		32		WM				
AR	Tan (26)	50	Moderate	SPGs	ER, RQLQ	4 weeks	Not mentioned	Not mentioned
		50		WM				
AR	Li (27)	27	Moderate	BSPGs	TNSS, VAS ventilation, eNO	4 weeks	In the BSPGs group and the CA group, one patient from each dropped out, while two patients withdrew from the WM group. None of these cases were included in the statistical analysis	Not mentioned
		27		WM				
		27		CA				
AR	Hu (28)	35	Moderate	BSPGs	SSS, RQLQ, ER	4 weeks	BSPGs group: Five cases dropped out. SPGs group: Three cases dropped out. CA group: Two cases dropped out. All were excluded. Adverse reactions: SPGs group: One case developed subcutaneous hematoma, significant improvement was observed after 1 week	Not mentioned
		35		SPGs				
		35		CA				
AR	Feng (29)	35	Moderate	SPGs	SSS, ER	4 weeks	Not mentioned	Not mentioned
		35		WM				
AR	Kan (30)	50	Moderate	SPGs	TNSS, TNNSS, RQLQ	4 weeks	Both groups had no severe complications after acupuncture. In the SPGs group, two patients had minor swelling in the jaw area post-acupuncture, resulting in a 4.0% adverse reaction rate. In the CA group, eight patients reported general fatigue, leading to a 16.0% adverse reaction rate. The difference in adverse reaction rates between the two groups was significant	Not mentioned
		50		CA				
AR	Li (31)	50	Moderate	SPGs	IgE, ER, EOC	30 days	Not mentioned	Not mentioned
		50		WM				
AR	Hou (32)	30	Moderate	SPGs	IgE, ER, EOS	2 weeks	Not mentioned	Not mentioned
		30		WM				
AR	Zhang (33)	25	Low	SPGs	SSS, TNSS, TNNSS, RQLQ	4 weeks	In the CA group, no adverse reactions were observed. In the SPGs group, one case had a burning sensation in the mouth, followed by bruising under the eye. Another case had local bruising, and five cases had temporary stinging, all of which resolved quickly	Not mentioned
		25		WM				
AR	Xu (34)	40	High	SPGs	ER, RQLQ, VAS ventilation	4 weeks	A total of 11 patients dropped out, with 5 in the SPGs group and 6 in the control group	1 month
		40		CA				
AR	Chen (35)	30	Moderate	SPGs	RQLQ, SSS	4 weeks	Not mentioned	3rd month, 6th month
		30		CA				
		30		WM				
AR	Fan (36)	16	Moderate	SPGs	RQLQ, TNSS, ER	6 weeks	During the treatment process, there were a total of 9 dropouts across all groups, with 3 in the SPGs group, 2 in the TCM group, 3 in the CA-TCM group, and 1 in the BC group. These dropouts were mainly due to the inability to adhere to a 6-week treatment schedule because of factors like living in another location, busy work, or academic commitments. There were no instances of facial bruising or needle fainting during the treatment, and no adverse reactions occurred	Not mentioned
		21		TCM				
		22		CA-TCM				
		21		BC				
AR	Wang (37)	60	Moderate	SPGs-CA	SSS, ER	18 days	Not mentioned	Not mentioned
		60		SPGs				
AR	Fu (38)	36	Low	SPGs	RQLQ, ER	4 weeks	Not mentioned	Not mentioned
		36		WM				
AR	Chen (39)	20	Moderate	SPGs	SSS	3 weeks	Out of the 45 participants initially enrolled, 6 dropped out during the trial, with 2 dropouts in the SPGs group and 4 dropouts in the CA group. During the trial, one patient in the treatment group experienced localized bruising on the cheek area after acupuncture. No significant adverse reactions were observed in the control group	Not mentioned
		19		CA				
AR	Li (40)	30	High	SPGs	Severe lateral nasal resistance value, ER	3 weeks	A total of 60 patients with chronic simple rhinitis were initially enrolled. One participant from the control group dropped out, resulting in a final cohort of 59 patients for analysis	Not mentioned
		29		CA				
AR	Sha (41)	35	Moderate	SPGs	RQLQ, TNSS, ER, General symptoms of VAS	4 weeks	One participant each from the SPGs group and the WN group dropped out during the study, resulting in a final cohort of 34 patients in each group, with a total of 68 patients participating and completing the study. The dropout rate was less than 10%. During the treatment period, no adverse reactions were observed in the SPGs group. In the WM group, one participant reported mild headache and discomfort, which completely resolved after discontinuing the treatment for 1 day	Not mentioned
		35		WM				
		48		CA				
AR	Zhang (42)	48	High	SPGs	RQLQ, TNSS, TNNSS, IgE, EOS, symptom days, waiting time	4 weeks	One patient in the SPGs group withdrew from the study due to lower eyelid bruising that occurred on the second day after treatment. In the CA group, six cases of subcutaneous bruising were observed, all of which disappeared on the second day without any special treatment	1 month
		48		CA				
AR	Mi (43)	33	High	SPGs	RQLQ, TNSS, scores for sneezing, rhinorrhea, nasal congestion, and nasal itching	4 weeks	In the clinical trial, nine patients experienced transient syncope during the initial treatment but recovered with rest. This symptom did not reoccur during subsequent acupuncture treatments. In the traditional acupuncture group, four cases presented with localized swelling and pain. These symptoms were alleviated with ice application and disappeared within 2 days	1 year
		36		CA				
		35		WM				
IS	Bornstein (44)	153	High	SPGs	Primary outcome: 3-month mRS improvement better than expected. Secondary outcomes: Improved aphasia and substantial neurological recovery at 3 months, 3-month stroke-related quality of life, self-reported function at 6 and 12 months, functional independence (mRS 0–2) at 3 months, distribution of disability outcomes at 3 months. Safety: Comparison of adverse events between active and sham groups at 3 months	5 days	No severe adverse effects related to stimulation occurred. There were no cases of symptomatic intracranial bleeding due to SPGs. Both groups showed similar rates of decreased neurological function (9.9%). Two severe adverse events were linked to the implant but resulted in full recovery. These events (0.6%) were associated with or possibly related to the implant: one case involved a nosebleed, and the other a torn extraction thread, which required surgical removal of the implant. Thankfully, both patients fully recovered	Follow-up assessments were conducted at 30, 60, and 90 days, including vital sign evaluations, general medical condition checks, adverse event monitoring, and functional outcome scale ratings
		100		SS				
IS	Bornstein (45)	555	High	SPGs	The primary outcome was a 3-month mRS score improvement assessed by masked evaluators. Additional 3-month outcomes included functional independence, self-care, stroke-related quality of life, and disability. Safety analysis considered adverse events related to implant procedures, removal, or stimulation, including serious adverse events, neurological deterioration, and mortality	5 days	In the safety analysis of 1,055 patients undergoing sphenopalatine ganglion stimulator placement (536 in the SPGs group and 519 in the SA group), no significant differences were found in the four main safety measures between the two groups	Follow-up assessments were conducted at 30, 60, and 90 days
		523		SS				
CH	Goadsby (46)	45	High	SPGs	Pain relief, pain disappearance within 15 min, and 1-hour pain relief maintenance. Response rates within 15 min, response rates for pain disappearance within 15 min, response rates for 1-h pain relief maintenance, weekly attack response rates. Adverse reactions	4 weeks	SPGs: 3 dropouts, SA group: 2 dropouts. A total of 9 severe adverse events were reported. Among these, three were related to the implant procedure (inhalation during intubation, nausea and vomiting, and vein damage or injury). One severe adverse event was an infection caused by the stimulation device and implant procedure. The other five severe adverse events were unrelated. No unexpected severe adverse events occurred	1 year
		48		SA				
CH	Schoenen (47)	28	High	SPGs	Pain relief within 15 min, pain disappearance after 15 min, attack frequency, acute response, frequency response, quality of life scales (HIT-6, SF-36v2 physical (PCS) and mental (MCS)), SAE safety endpoints	3–8 weeks	There were no dropouts. There were five cases of serious adverse events related to the device or procedure	1 year
		28		SS				
		28		substim				
PTN	Zhao (48)	30	Moderate	SPGs	ER, VAS pain score	3 weeks	Not mentioned	Not mentioned
		30		CA				
PTN	Liu (49)	30	Low	SPGs	ER, VAS pain score	21 days	Not mentioned	Not mentioned
		30		CA				
PTN	He (50)	33	Moderate	SPGs	ER, Time, frequency, and intensity of pain attacks	25 days	One case dropped out of each group due to the patient's own reasons, none adverse events occurred	Not mentioned
		32		CA				
NNCF	Wang (51)	25	Moderate	SPGs	VAS pain score, SP, VIP, NPY	24 h	None	None
		25		SA				
NNCF	Wang (52)	25	Moderate	SPGs	Self-reported nasal airflow changes, nasal patency assessment, nasal exhaled nitric oxide measurement	24 h	None	None
		25		SA				
NNCF	Wang (53)	20	High	SPGs	All subjects were observed for nasal airflow, NAR, NCV, eNO, and neuropeptides SP, VIP, and NPY in nasal secretions at baseline, 30 min post-acupuncture, 2 h, and 24 h	24 h	None	None
		19		SA				
PCSO	Chen (54)	48	Moderate	SPGs	ER, pure-tone audiometry in the affected ear, and tympanometry in the affected ear	8 weeks	Not mentioned	Not mentioned
		47		WM				
RFP	Luo (55)	20	Moderate	SPGs	Sunnybrook (Toronto) facial nerve score, House-Brackmann (H-B), ER	15 days	Not mentioned	Not mentioned
		22		CA				
CTTH	Wang (56)	50	Moderate	SPGs	ER, headache attack frequency, attack duration, severity and associated symptom scores, GQOLI-74 questionnaire on physical, psychological, social, and material wellbeing	3 weeks	Not mentioned	Not mentioned
		50		WM				
LF-SPGs in CCH	Barloese (57)	16	Moderate	SPGs	Significant differences in HRV parameters such as HR, SDNN, SD2, and HFnu	2 days	5 patients excluded due to various reasons, More patients experienced cranial autonomic symptoms (CAS) during LF-SPGs compared to SS	One day after discharge
LF-SPGs in CCH	Guo (58)	20	High	SPGs	headache intensity, cephalic autonomic symptoms (CAS), mechanical perception, pain thresholds, blood pressure, heart rate, and specific blood markers	2 days	Not mentioned	One day after the stimulation ends
		SS						

**Figure 2:**
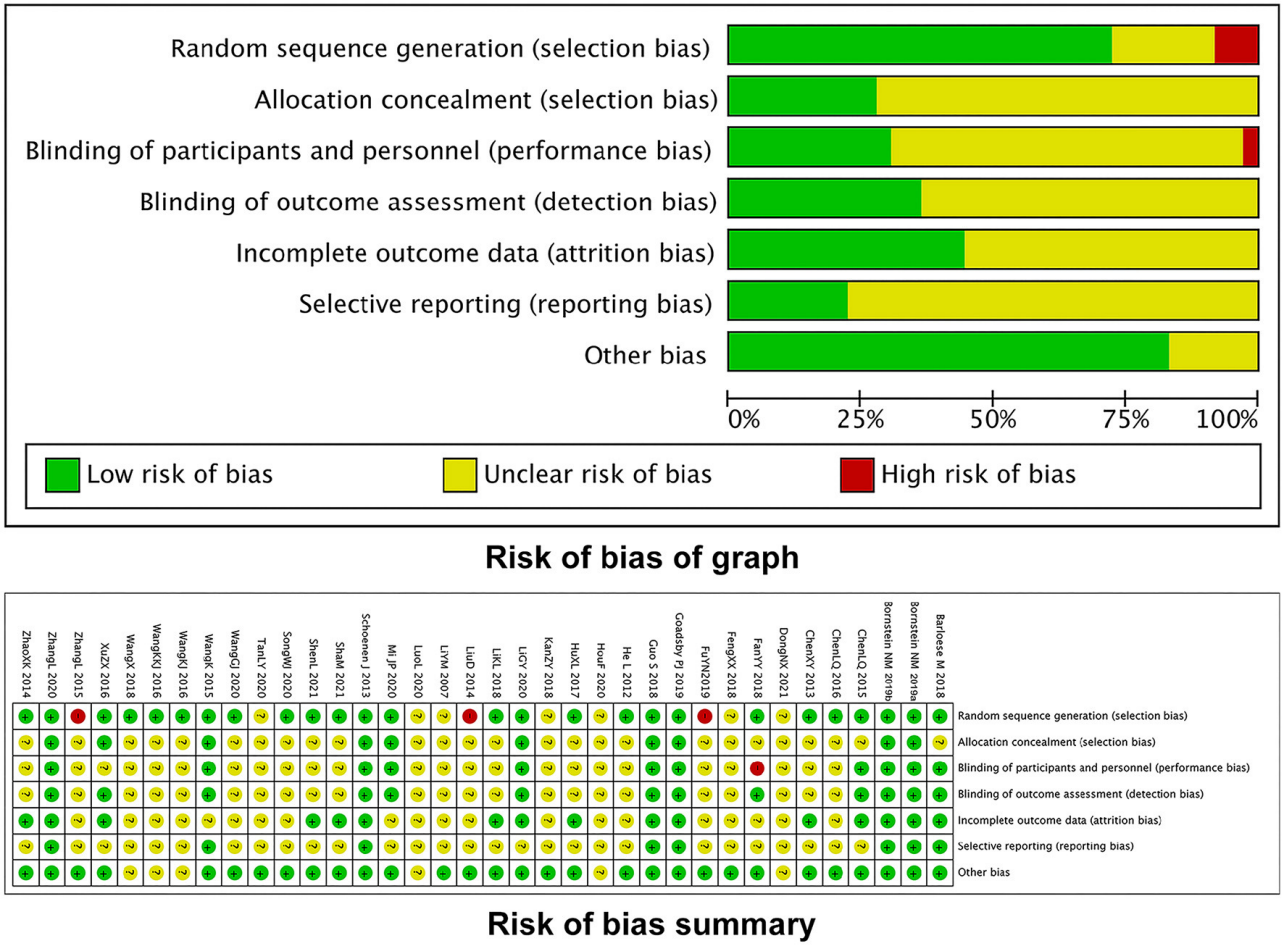
Map for ROB.

We assessed the quality assessment by the Cochrane Handbook for Systematic Reviews of Interventions. There were 10 high quality (34, 40, 42–47, 53, 58), 23 moderate quality, and three low quality (33, 38, 49). The ROB map is shown in Figure 2.

### 3.2 Allergic rhinitis, AR

This study evaluated the efficacy of SPGs in AR using five key metrics: the Rhinoconjunctivitis Quality of Life Questionnaire (RQLQ), Total Nasal Symptom Score (TNSS), Symptom and Sign Score (SSS), Effective Rate (ER), and Immunoglobulin E (IgE). RQLQ was utilized as an outcome measure in 13 articles (23, 24, 26, 28, 30, 33–36, 38, 41–43). TNSS was employed as an outcome measure in seven articles (27, 30, 33, 36, 41–43). SSS was utilized as an outcome measure in eight articles (23, 24, 28, 29, 33, 35, 37, 39). ER was used as an outcome in 13 articles (23, 25, 26, 28, 29, 31, 32, 34, 36–38, 40, 41). IgE levels were assessed as an outcome in 4 articles (25, 31, 32, 42).

#### 3.2.1 Network meta-analysis

##### 3.2.1.1 RQLQ

In terms of decreasing RQLQ scores, SPGs outperformed both WM [–7.44 (–13.95, –0.93)] and CA [–12.68 (–19.06, –6.29)]. It's worth noting that the remaining comparisons did not demonstrate statistical significance. More detailed information can be found in Table 2A.

**Table 2A:** Network meta-analysis results of RQLQ.

	**SPGs**	**CA-TCM**	**TCM**	**BSPGs**	**BC**	**WM**
CA-TCM	0.42 (–15.20, 16.05)					
TCM	0.16 (–15.47, 15.80)	–0.26 (–15.92, 15.40)				
BSPGs	–1.10 (–17.54, 15.33)	–1.53 (–24.20, 21.15)	–1.27 (–23.95, 21.41)			
BC	–0.92 (–16.56, 14.72)	–1.34 (–17.01, 14.33)	–1.08 (–16.75, 14.59)	0.19 (–22.50, 22.87)		
WM	–7.44 (–13.95, –0.93)^*^	–7.87 (–24.79, 9.06)	–7.61 (–24.54, 9.33)	–6.34 (–23.77, 11.10)	–6.53 (–23.46, 10.41)	
CA	–12.68 (–19.06, –6.29)^*^	–13.10 (–29.97, 3.77)	–12.84 (–29.72, 4.03)	–11.57 (–27.98, 4.83)	–11.76 (–28.64, 5.12)	–5.24 (–13.38, 2.91)

Both MD and 95% CI were >0 or < 0, indicating a significant difference between the intervention measures (*P* < 0.05).

^*^Significant difference between the intervention measures.

##### 3.2.1.2 TNSS

In terms of decreasing TNSS scores, CA-TCM was superior to BSPGs [–1.81 (–3.33, –0.30)], Blank Control (BC) [–2.64 (–3.89, –1.39)], WM [–2.77 (–4.24, –1.30)], CA [–3.04 (–4.51, –1.58)]. SPGs was superior to BC [–1.52 (–2.88, –0.16)], WM [–1.65 (–2.26, –1.04)], CA [–1.92 (–2.52, –1.33)]. Traditional Chinese Medicine (TCM) was superior to BC [–1.55 (–2.90, –0.20)], WM [–1.68 (–3.24, –0.12)], CA [–1.95 (–3.51, –0.40)]. BSPGs was superior to WM [–0.96 (–1.50, –0.42)], CA [–1.23 (–1.73, –0.73)]. No other comparisons demonstrated statistical significance. Additional details are provided in Table 2B.

**Table 2B:** Network meta-analysis results of TNSS.

	**CA-TCM**	**SPGs**	**TCM**	**BSPGs**	**BC**	**WM**
SPGs	–1.12 (–2.46, 0.22)					
TCM	–1.09 (–2.42, 0.24)	0.03 (–1.40, 1.46)				
BSPGs	–1.81 (–3.33, –0.30)^*^	–0.69 (–1.41, 0.02)	–0.72 (–2.32, 0.87)			
BC	–2.64 (-3.89, –1.39)^*^	–1.52 (–2.88, –0.16)^*^	–1.55 (–2.90, –0.20)^*^	–0.83 (–2.36, 0.71)		
WM	–2.77 (–4.24, –1.30)^*^	–1.65 (–2.26, –1.04)^*^	–1.68 (–3.24, –0.12)^*^	–0.96 (–1.50, –0.42^*^	–0.13 (–1.62, 1.36)	
CA	–3.04 (–4.51, –1.58)^*^	–1.92 (–2.52, –1.33)^*^	–1.95 (–3.51, –0.40)^*^	–1.23 (–1.73, –0.73)^*^	–0.40 (–1.89, 1.08)	–0.27 (–0.77, 0.23)

Both MD and 95% CI were >0 or < 0, indicating a significant difference between the intervention measures (*P* < 0.05).

^*^Significant difference between the intervention measures.

##### 3.2.1.3 SSS

In terms of decreasing SSS scores, none of comparisons were statistically significant. Detailed information can be found in Table 2C.

**Table 2C:** Network meta-analysis results of SSS.

	**SPGs**	**SPGs-CA**	**WM**	**BSPGs**
SPGs-CA	–2.44 (–61.33, 56.44)			
WM	–5.27 (–34.22, 23.67)	–2.83 (–68.24, 62.58)		
BSPGs	–8.71 (–62.45, 45.03)	–6.27 (–85.64, 73.10)	–3.44 (–63.14, 56.26)	
CA	–19.46 (–46.84, 7.92)	–17.02 (–81.09, 47.05)	–14.19 (–49.75, 21.37)	–10.75 (–64.49, 42.99)

##### 3.2.1.4 ER

In terms of increasing ER, Network analysis reveals that there is no statistical significance observed in the comparison of various intervention measures. Further details are provided in Table 2D.

**Table 2D:** Network meta-analysis results of ER.

	**SPGs**	**BSPGs**	**WM**	**CA**	**TCM**	**CA-TCM**
BSPGs	1.01 (0.03, 36.93)					
WM	1.00 (0.25, 4.04)	0.99 (0.02, 47.40)				
CA	1.01 (0.10, 9.88)	1.01 (0.03, 36.93)	1.01 (0.07, 14.63)			
TCM	0.77 (0.01, 40.75)	0.76 (0.00, 162.85)	0.77 (0.01, 51.69)	0.76 (0.01, 73.97)		
CA-TCM	0.73 (0.01, 38.90)	0.73 (0.00, 155.49)	0.73 (0.01, 49.35)	0.73 (0.01, 70.62)	0.96 (0.02, 50.34)	
BC	0.77 (0.01, 40.75)	0.76 (0.00, 162.85)	0.77 (0.01, 51.69)	0.76 (0.01, 73.97)	1.00 (0.02, 52.73)	1.05 (0.02, 55.13)

#### 3.2.2 SUCRA curve

##### 3.2.2.1 RQLQ

**Figure 3:**
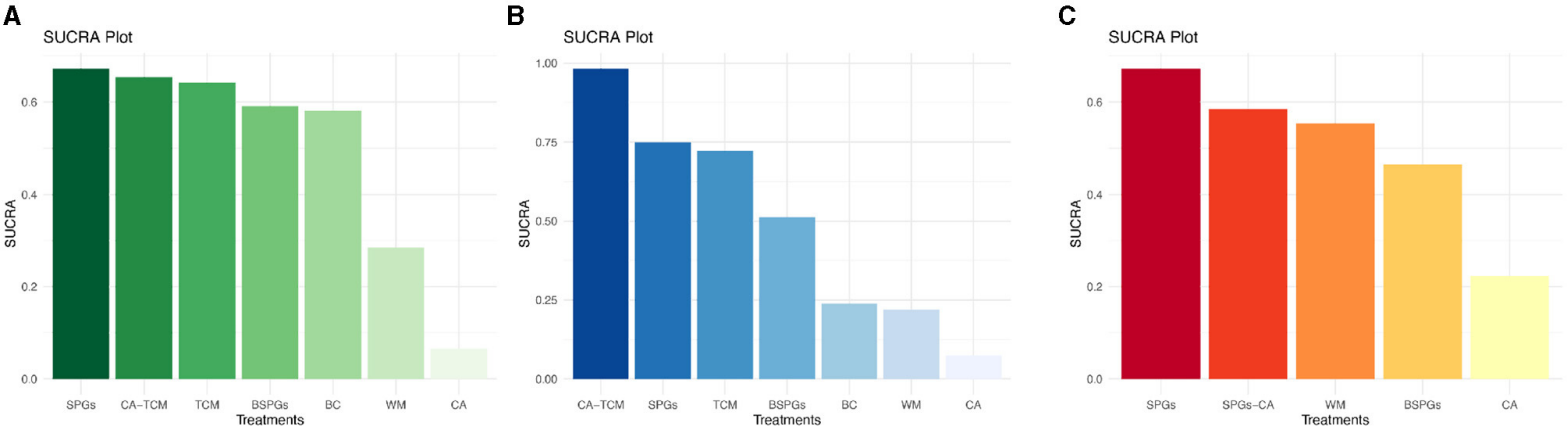
SUCRA Plot of RQLQ, TNSS, SSS. **(A)** Rhinoconjunctivitis quality of life questionnaire, RQLQ. **(B)** Total Nasal Symptom Score, TNSS. **(C)** Symptom and sign score, SSS.

The SUCRA curve ranked interventions as follows, from highest to lowest: SPGs (67.3%), CA-TCM (65.4%), TCM (64.2%), Bilateral sphenopalatine ganglion stimulation (BSPGs) (59.1%), BC (58.2%), WM (28.5%), and CA (6.6%). Further details are presented in Figure 3A.

##### 3.2.2.2 TNSS

The SUCRA curve ranked the interventions as follows, from highest to lowest: CA-TCM (98.3%), SPGs (74.9%), TCM (72.2%), BSPGs (51.3%), BC (23.9%), WM (22.0%), and CA (7.4%). Further details are presented in Figure 3B.

##### 3.2.2.3 SSS

The SUCRA curve rankings from highest to lowest are as follows: SPGs (67.3%), sphenopalatine ganglion stimulation Combined with Conventional Acupuncture (SPGs-CA) (58.5%), WM (55.4%), BSPGs (46.5%), and CA (22.3%). Further details are presented in Figure 3C.

#### 3.2.3 Meta analysis

##### 3.2.3.1 ER

**Figure 4:**
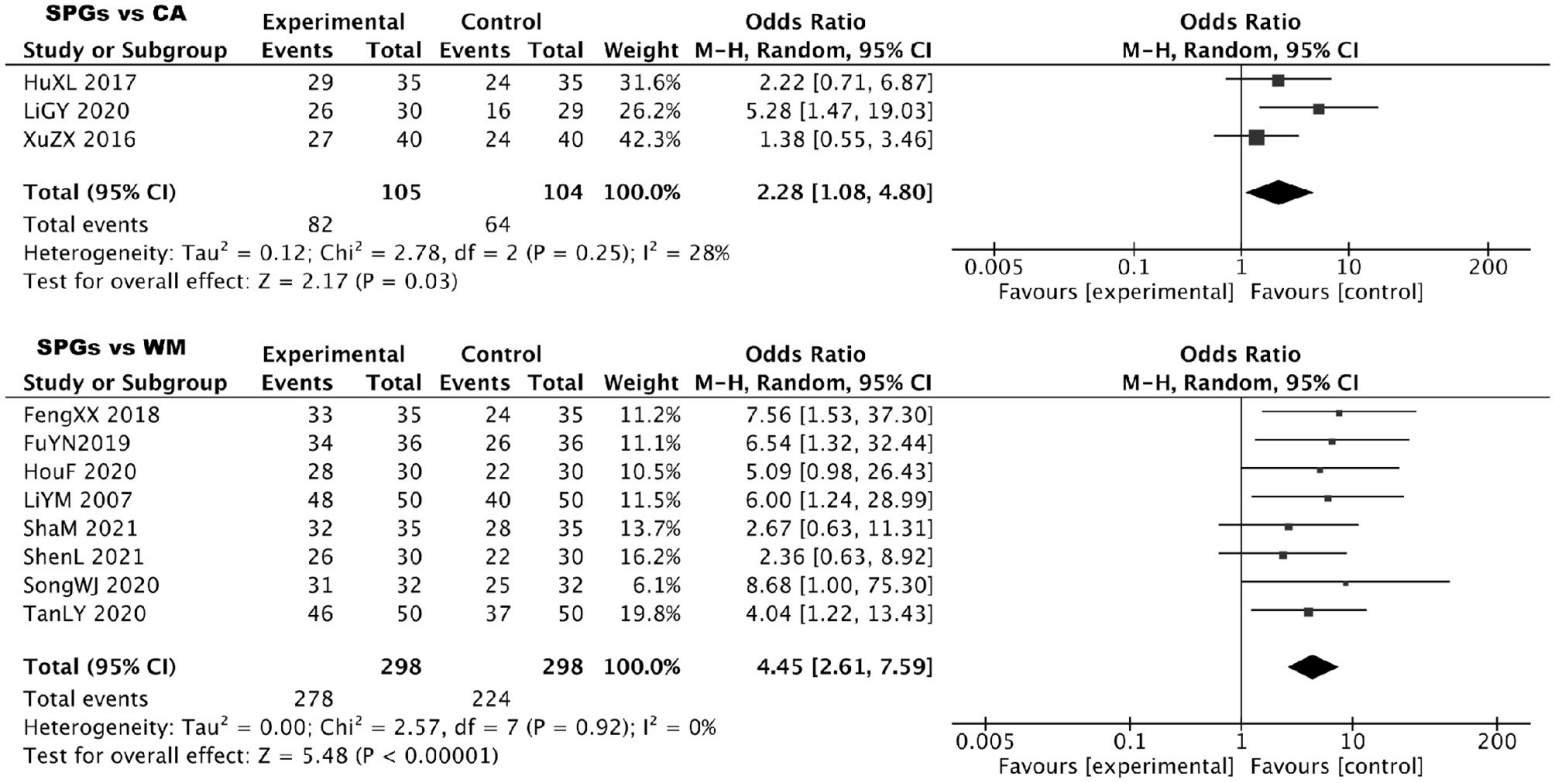
Forest plot for AR using SPGs: Efficacy rate as the outcome measure.

By comparing paired analyses of SPGs vs CA and SPGs vs WM, it was discovered that both Experimental group were superior to the Control group, namely, SPGs were superior to CA and SPGs were superior to WM, and the heterogeneity was small and the difference was statistically significant. Details are shown in Figure 4.

##### 3.2.3.2 IgE

In terms of reducing IgE, the results of all three investigations showed that SPGs was not statistically significant when compared to WM. Further details are presented in Supplementary Figure S1.

### 3.3 Ischemic strokes, IS

Two RCTs investigating the use of SPGs in treating IS were included. In the Impact-24A (44) and Impact-24B (45) studies, the implementation of SPGs involved the implantation of a neurostimulator electrode under local anesthesia. This electrode, measuring 23 mm in length and 2 mm in diameter, was precisely inserted into the pterygopalatine canal, closely adjacent to the SPG. In the Impact-24A study (44), a total of 303 participants underwent implantation and entered the randomization phase. Among them, 253 individuals [153 in the SPGs group and 100 in the sham stimulation (SS) group] received at least one stimulation session and were thus included in the mITT dataset. In the mITT population, a comparison between SPGs and SS revealed that 49.7% of participants in the SPGs group and 40% in the SS group surpassed the expected improvement in the primary efficacy endpoint. The odds ratio (OR) was 1.48 (95% CI: 0.89–2.47), with a *P*-value of 0.13, indicating that the difference was not statistically significant. However, a subgroup analysis among individuals with CCI revealed that 50% in the SPGs group and 27% in the SS group showed improvement beyond expectations in the primary efficacy endpoint. The OR was 2.70 (95% CI: 1.08–6.73), with a statistically significant *P*-value of 0.03, demonstrating that SPGs is potentially more effective in enhancing recovery from cortical damage than SS.

In the Impact-24B study (45), 1,078 patients were enrolled, and 1,000 received at least one session of SPGs or SS, entering the mITT population (481 in the SPGs group and 519 in the SS group). In the efficacy comparison, 49% (234 out of 481) in the SPGs group and 45% (236 out of 519) in the SS group showed improvement exceeding expectations in the primary efficacy endpoint, resulting in an OR of 1.14 (95% CI: 0.89–1.46), with a *P*-value of 0.31. However, in the subgroup with CCI, 50% (121 out of 244) in the SPGs group and 40% (110 out of 276) in the SS group exceeded the expected improvements, yielding an OR of 1.48 (95% CI: 1.05–2.10) with a statistically significant *P*-value of 0.0258. The Hochberg procedure was applied, indicating neutral results for the overall mITT population and positive outcomes for the CCI subgroup. A dose-response relationship was identified through retrospective multivariable logistic regression analysis using a restricted cubic spline model, which showed an inverted U-shaped curve (*P* = 0.0034). Clinical outcomes indicated improvements with moderate and low levels of SPGs, while high stimulation levels did not yield significant clinical improvements.

**Figure 5:**
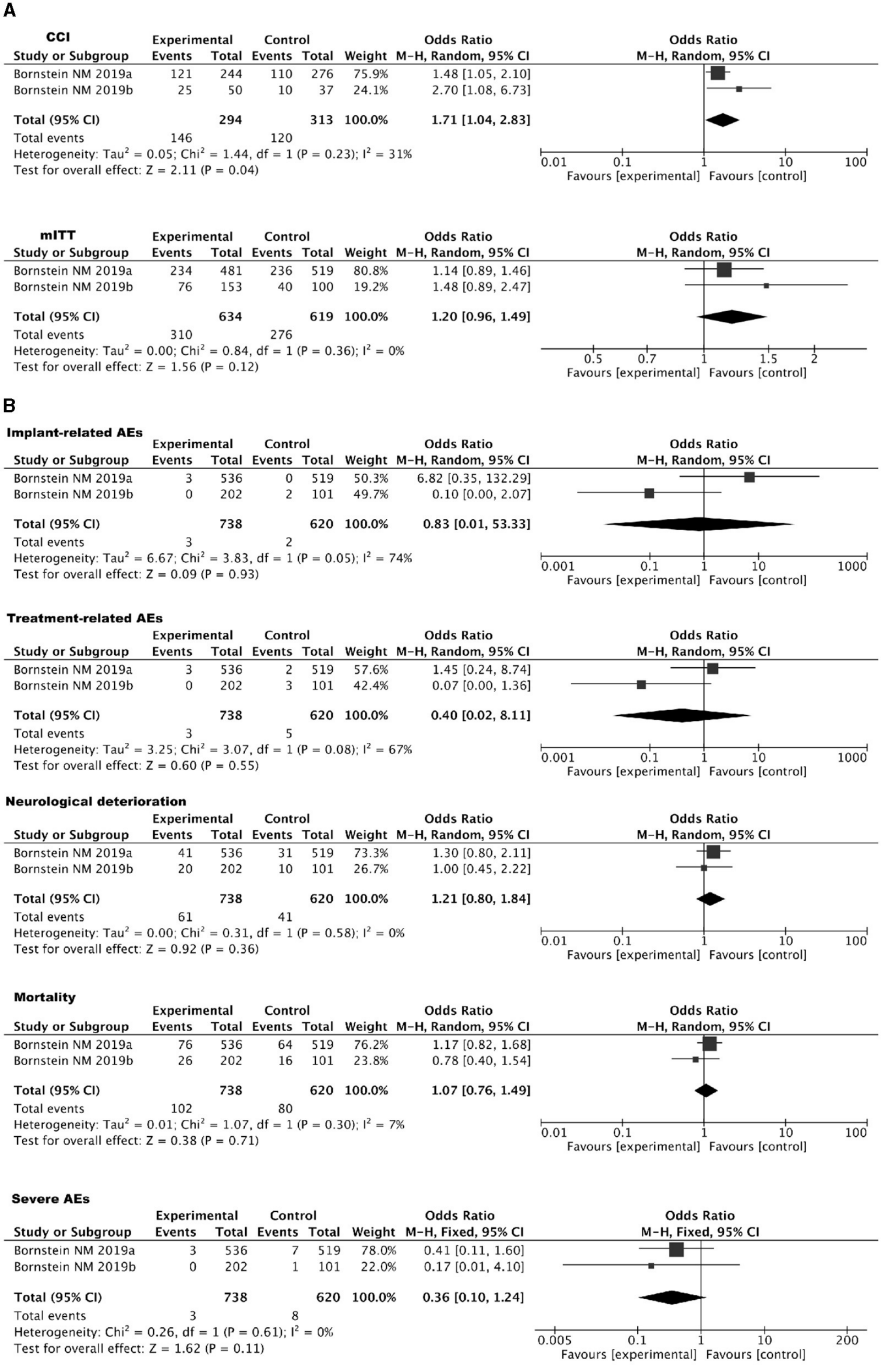
**(A)** Forest plot for IS using SPGs: mITT and CCI populations. **(B)** Forest plot of safety analysis: SPGs in IS.

Regarding the primary outcome measure of the two RCTs, which pertains to mRS improvement beyond expected values within 3 months, participants were categorized into two groups for comparative analysis: the mITT and CCI groups. Detailed results are presented in Figure 5A. Safety analyses were conducted in both trials, and in both cases, the *P*-values exceeded 0.05. This suggests that adverse reactions did not reach statistical significance. Detailed results can be found in Figure 5B.

### 3.4 Cluster headache, CH

Two RCTs were included in the assessment of SPGs for treating CH (46, 47). In the studies reviewed, participants underwent implantation of the Autonomic Technologies Inc. SPG Neurostimulation System under general anesthesia. This device, comprising a miniaturized implant with a built-in lead containing six stimulating electrodes, was positioned adjacent to the SPG within the PPF. The primary outcome measure focused on the number of participants experiencing relief within 15 min of starting stimulation, while the secondary outcome measures the number of individuals whose pain disappeared within the same timeframe. The data reveal that SPGs treatment led to statistically significant improvements in both headache relief and disappearance within 15 min of starting the treatment, as evidenced by different levels of effectiveness across the studies. Despite high heterogeneity (*I*^2^≥95%) indicating variation between study outcomes, individual studies like Goadsby (59) and Schoenen (18) demonstrate significant effects, with overall analyses yielding a statistically significant positive impact (*P* = 0.002) on CH symptoms. Hence, the evidence suggests that SPGs treatment may have a beneficial effect on alleviating and resolving headaches, warranting careful consideration of its application due to study variability. Further research with standardized methodologies is required to confirm these findings. Detailed results are presented in Supplementary Figure S2.

### 3.5 Analysis of LF-SPGs in CCH

Two RCTs investigated LF-SPGS in CCH. In these studies (57, 58), the PulsanteTM SPG Neurostimulator was locally implanted near the SPG. One trial explored LF-SPGS's effects on Heart Rate Variability (HRV) in 16 CCH patients, revealing significant differences in HRV indices such as heart rate, SDNN, SD2, and HFnu between LF-SPGS and SS. Specifically, LF-SPGS initially elevated heart rate, indicating increased sympathetic activity, and subsequently heightened parasympathetic activity, with six out of 10 reported cluster-like headache attacks occurring post LF-SPGS. Another study involving 20 CCH participants assessed various parameters including headache intensity, CAS, and others, finding 35% of patients experienced cluster-like attacks post LF-SPGS with no significant differences in headache intensity between LF-SPGS and SS groups. Notably, 80% reported CAS post LF-SPGS, a significantly higher rate than SS, but no notable changes in mechanical pain thresholds or other measured variables were observed. Collectively, these studies illuminate LF-SPGS's impact on autonomic functions and provide insights into CH's pathophysiology, suggesting that while LF-SPGS can elicit autonomic symptoms, it does not necessarily trigger CH attacks, indicating the necessity of deeper brain structure involvement in CH pathogenesis.

### 3.6 Primary trigeminal neuralgia, PTN

Three RCTs were included to evaluate the effectiveness of SPGs in treating PTN (48–50). The primary outcome measures included improvements in headache intensity, assessed using the Visual Analog Scale (VAS), and an overall evaluation of effectiveness.

The comprehensive data analysis shows that SPGs have statistically significant improvements in headache intensity (*P* < 0.05), with an overall mean difference of –0.97 [95% CI: (–1.51, –0.42)], indicating that the experimental group has significantly improved compared to the control group. Moreover, heterogeneity is very low (*I*^2^ = 0%), indicating consistent results across studies. Detailed results are available in Supplementary Figure S3a.

Regarding the ER improvement between SPGs and CA, the overall OR is 2.18 [95% CI: (0.78, 6.08)], showing no statistically significant difference, indicating that there is no significant difference in the event occurrence rate between the experimental and control groups. Heterogeneity is also very low (*I*^2^ = 0%), indicating that the study results are consistent. Detailed results are available in Supplementary Figure S3b.

### 3.7 Normal nasal cavity function, NNCF

Three RCTs were conducted to assess the effect of SPGs on NNCF (51–53). Outcome measures were evaluated at three specific time points: 30 min, 2 h, and 24 h after stimulation. In these studies across three different time points, the experimental group showed significant improvements over the control group in reducing NAR, increasing NCV, modulating eNO, and elevating the levels of SP, VIP, and NPY. The results indicate that the experimental treatment had significant short-term and long-term effects on these indicators (*P* < 0.05). Additionally, the heterogeneity among all indicators at each time point was generally low, suggesting consistency in the research results. This demonstrates that the experimental intervention has a consistent effect on these biomarkers, both in the short term and extending up to 24 h later. Detailed results are available in Supplementary Figures S4a–c.

### 3.8 SPGs effects on pediatric chronic secretory otitis, refractory facial paralysis, and chronic tension-type headache

One of the included RCTs investigated the use of SPGs in treating PCSO (54). The study involved 95 children diagnosed with chronic secretory otitis media, who were randomly assigned to either the SPGs group or a drug group (comprising orally administered Myrtle oil enteric-coated capsules and mometasone furoate nasal spray). Both groups followed an 8-week treatment protocol during which the research team assessed the intervention's effectiveness. Evaluations took place before the start of treatment and 3 months after completion, with a focus on the effectiveness rate, pure tone audiometry test results for the affected ear, and tympanic acoustic admittance test results for the affected ear. The data revealed statistically significant improvements in all three parameters (*P* < 0.05).

In the collected literature, we identified a single RCT focused on RFP (55). This study included a total of 42 patients who were subsequently divided into two groups: the SPGs group and the CA group. Both groups underwent a 15-day treatment regimen. After the treatment period, assessments were conducted to evaluate the Sunnybrook (Toronto) facial nerve score, H-B facial nerve function grade, and overall treatment effectiveness in both groups. The findings revealed that, following treatment, the Sunnybrook (Toronto) facial nerve score, H-B facial nerve function grade, and overall treatment efficacy in the SPGs group exceeded those in the CA group, with a statistically significant difference (*P* < 0.05).

In the included literature, we found one RCT focused on CTTH (56). his study involved a cohort of 100 cases, which were divided into two groups: the SPGs group and the oral ibuprofen sustained-release capsule group. The treatment duration for all patients was 3 weeks. The study assessed changes in physical function, psychological function, social function, and material life scores using the General Quality of Life Inventory-74 (GQOLI-74) before and after treatment. The results indicated that the SPGs group achieved a higher efficacy rate compared to the drug group. After treatment, both groups showed significant reductions in the frequency, duration, severity, and accompanying symptom scores compared to pre-treatment levels (*P* < 0.05). Furthermore, the SPGs group's scores in these aspects were significantly lower than those in the drug group (*P* < 0.05). Additionally, post-treatment assessments revealed a significant improvement in physical function, psychological function, social function, and material life scores for both groups based on the GQOLI-74 questionnaire (*P* < 0.05), with the SPGs group outperforming the drug group (*P* < 0.05).

## 4 Discussion

### 4.1 Evidence synthesis

This study systematically reviewed 36 articles, focusing on RCTs related to SPGs. The diseases examined included AR, IS, CH, PTN, NNCF, the effects of SPGs on PCSO, RFP, and CTTH. Additionally, the study analyzed the impact of LF-SPGs on CCH. Subsequent quality assessments categorized the studies into high, medium, and low-quality studies, ensuring the reliability of the results. Detailed data extraction was performed for each disease, assessing symptom improvements, enhancements in quality of life, and physiological indicators. The study concluded with a meta-analysis evaluating the therapeutic effects of SPGs across diseases, and statistical analysis compared intervention outcomes. This systematic approach provides a comprehensive understanding of the efficacy of SPGs, guiding future clinical practices and research. This article categorizes studies according to the method of SPGs employed: electrode implantation and acupuncture needle stimulation. Specifically, studies Bornstein (44, 45), Goadsby (46), Schoenen (47), Barloese (57), and Guo (58) used implanted electrodes for stimulation, while the other studies employed acupuncture needle stimulation.

#### 4.1.1 AR

This study comprises an analysis of 21 literature pieces that investigate the treatment of AR, encompassing four high-quality, two low-quality, and fifteen medium-quality papers. SPGs outperform CA and WM in improving RQLQ scores. CA-TCM leads in TNSS improvement, with no clear advantage in SSS and IgE levels. SPGs also excel in ER enhancement, though overall differences are minimal. These results closely align with clinical outcomes and are supported by numerous case reports and systematic reviews confirming the efficacy of SPGs in treating AR. Characterized by chronic inflammation of the nasal mucosa, primarily driven by IgE mediated responses. The development of AR involves essential roles of hyperreflexia within the nasal mucosa and dysfunction of nasal nerves (60). Tracey (61) introduced the “inflammatory reflex,” highlighting how the nervous system significantly influences immune responses. This system adjusts inflammatory reactions promptly, similar to its regulation of heart rate and other essential physiological parameters. The nasal cavity's defense mechanism is intricately linked to the functions of the trigeminal and sphenopalatine ganglia, which are crucial for regulating sensations, vascular responses, and glandular secretions within the nasal mucosa. Notably, the parasympathetic nervous system significantly affects on the nasal mucosa, impacting both healthy and pathological states (62). External environmental changes or internal condition alterations can trigger neurogenic inflammation, leading to AR symptoms, including sneezing, rhinorrhea, and nasal congestion (63). Furthermore, the neurological influences on the nasal mucosa span cellular and molecular dimensions, involving interactions among inflammatory mediators, cytokines, neuropeptides, and the structural components of nerves, blood vessels, and glands within the nasal mucosa (64). Howarth (65) emphasizing the importance of understanding and modulating these pathways are vital for the effective treatment and management of AR.

#### 4.1.2 NNCF

This section explores the impact of SPGs on the NNCF through an analysis of three selected studies. These studies, consisting of one high-quality and two medium-quality research papers, collectively show SPGs significantly improve NNCF factors like NAR, NCV, eNO, SP, VIP, and NPY compared to SA. This indicates that healthy volunteers treated with SPGs can increase sympathetic nerve excitability, contributing to improved nasal ventilation. The immunomodulatory effects of SPGs on the nasal mucosa can be attributed to diminished SP and neuropeptide release, thereby mitigating IgE-mediated allergic responses and improving the condition of the nasal cavity (20). Upon nerve stimulation, the resulting impulse bifurcates: one branch reaches the peripheral nerve endings to affect the target organs, while the other transmits signals to the central nervous system, culminating in integration within the hypothalamus. This process modulates central sensitization, alters neurological functions, and thereby exerts a regulatory effect of the nervous system on AR (66).

#### 4.1.3 IS

This research evaluates two high-quality publications addressing IS treatments. Impact-24A and Impact-24B trials show that SPGs and SS are similarly effective overall, but SPGs are more effective for CCI. Moderate SPGs stimulation yields the best results, with minimal side effects. Extending beyond these studies, additional research underscores the potential of SPGs in dilating cortical arterioles, fostering reperfusion in ischemic areas, augmenting ipsilateral cerebral blood flow, reducing infarct size (13, 67), preserving the blood-brain barrier (15), and improve neurological function (7). Furthermore, recent single-arm trials suggest the feasibility, safety, and potential efficacy of SPGs administered within 24 h post-stroke, particularly in patients with cortical involvement (68). The neurobiological mechanisms underpinning the efficacy of SPGs in IS treatment encompass four primary areas: (1) Reperfusion: The SPG exerts a direct influence on cerebral vessels via its parasympathetic fibers, facilitating vasodilation independently of metabolic demands and perfusion pressure, thereby modulating cerebral blood flow (CBF). The secretion of parasympathetic neurotransmitters, such as NO and VIP, induces vasodilation and enhances blood flow (59). In the Impact-24A and Impact-24B trials, particularly among patients with CCI, SPGs was associated with notable improvements in global disability, underscoring its potential to augment CBF and aid in stroke recovery. (2) Blood-Brain Barrier (BBB) Stability: Parasympathetic innervation of cerebral vessels plays a pivotal role in regulating the BBB. NO, released by postganglionic parasympathetic fibers, is capable of stabilizing and restoring BBB integrity under specific conditions. Such stabilization mitigates post-ischemic brain edema and consequent damage (69, 70). The Impact-24A and Impact-24B trials furnished evidence that BBB stabilization, likely facilitated by enhanced CBF and direct effects of SPGs, contributes to therapeutic benefits by ameliorating post-ischemic brain injury. (3) Neuroprotection: Activation of the SPG triggers central cholinergic and adrenergic pathways, conferring anti-inflammatory, anti-apoptotic, and anti-excitotoxic effects that safeguard neurons, glial, and endothelial cells within ischemic contexts (71). The Impact-24A and Impact-24B trials revealed that SPGs offers substantial advantages for individuals with cortical involvement, pinpointing those most likely to derive acute neuroprotective benefits from such intervention. (4) Enhanced neuroplasticity and neurogenesis: Stimulation of the SPG fosters the reorganization of neural networks and the proliferation of new neurons, bolstering functional recovery in regions compromised by stroke. This phenomenon, linked to increased neuroplasticity in the perilesional zone and the contralateral homotopic cortex, is essential for recuperation following a stroke (72–75). The methodology and timing of the Impact-24A and Impact-24B trials accentuate the instrumental role of SPGs in facilitating neuroplasticity, neurogenesis, and neural repair post-stroke.

#### 4.1.4 CH

This study involves two high-quality RCTs. Despite high heterogeneity (*I*^2^≥95%) indicating variation between study outcomes, individual studies like Goadsby (59) and Schoenen (18) demonstrate significant effects, with overall analyses yielding a statistically significant positive impact (*P* = 0.002) on CH symptoms. Hence, the evidence suggests that SPGs treatment may have a beneficial effect on alleviating and resolving headaches, warranting careful consideration of its application due to study variability. Further research with standardized methodologies is required to confirm these findings. Various approaches, including blockade (76), radiofrequency (77), excision (78), and implantation of stimulators (47, 79), have been explored in the treatment of CH through SPGs. This suggests a crucial role of the SPG in managing CH. Studies have indicated that pharmacological blockade of the SPG yields significant therapeutic effects for CH. While excision of the ganglion and radiofrequency techniques provide some relief, these interventions incur irreversible damage to the SPG. In contrast, the application of neuromodulation techniques holds promising prospects, demonstrating notable efficacy in RCTs, open studies, and follow-up assessments (80). During CH attacks, the parasympathetic system exhibits active behavior, indicating its significant role in the pathophysiology of CHs. Specifically, the activation of the parasympathetic system is associated with the pain and cranial autonomic symptoms during CH attacks. The principle of SPGs is based on interrupting the SPG, aiming to disrupt the interaction between the parasympathetic and trigeminal systems. By altering parasympathetic conduction, SPGs seeks to alleviate or prevent CH attacks. However, the exact mechanism of action of SPGs remains unclear. SPGs typically involves high-frequency stimulation. Such stimulation could lead to neurotransmitter depletion, causing a physiological block. This might explain why high-frequency stimulation produces immediate effects during the acute phase of CHs. Moreover, repeated stimulation could exert long-term modulatory effects on the parasympathetic system through induced neuroplastic changes (81).

#### 4.1.5 Analysis of LF-SPGs in CCH

Two articles examine if LF-SPGs cause CH (one high-quality, one medium-quality). Managing CCH may require addressing autonomic dysregulation and deeper neural processes. The active role of the parasympathetic system during CH episodes underscores its vital contribution to the disorder's pathophysiology, evident through its link to the pain and cranial autonomic symptoms observed during attacks. The rationale behind SPGs is grounded in its ability to disrupt the SPG, aiming to halt the interaction between the parasympathetic and trigeminal systems. Through the modulation of parasympathetic transmission, SPGS seeks to mitigate or prevent CH episodes (81).

#### 4.1.6 PTN

The study of PTN involves three articles, with one being of low-quality and two of medium-quality. The comprehensive data analysis indicates that while SPGs significantly reduce headache intensity compared to the control group (*P* < 0.05), there is no statistically significant difference in the efficacy rate between SPGs and CA. The SPG, an important nerve node closely related to the trigeminal nervous system, is involved in the transmission of facial pain sensations. By administering SPGs, it is possible to directly interrupt or attenuate the transmission of pain signals from the face to the brain's pain center, thus reducing or eliminating the pain caused by PTN. Patients with PTN often experience symptoms of autonomic nervous system activation during pain attacks, such as increased tear production and nasal congestion. SPG contains parasympathetic nerve fibers, and SPGs help alleviate these autonomic nervous system symptoms by modulating the activity of the parasympathetic nervous system.

#### 4.1.7 SPGs effects on PCSO, RFP, and CTTH

A medium-quality RCT has shown that SPGs significantly enhances outcomes for PCSO, as indicated by improved ear audiometry and tympanogram results. Similarly, another RCT of comparable quality revealed that SPGs are superior in treating RFP, evidenced by notable improvements in Sunnybrook and House-Brackmann (H-B) facial nerve scores relative to CA. Furthermore, SPGs have been found to outperform WM in managing CTTH, according to another medium-quality RCT. Despite these findings, there is a notable gap in mechanistic research regarding SPGs' application in PCSO, RFP, and CTTH treatments. Consequently, further investigations are essential to validate these findings and elucidate the mechanisms through which SPGs exert their therapeutic effects in these conditions.

### 4.2 Limitations analysis

This study is confined to RCTs, consequently omitting a wide array of significant literature. Such a selection criterion might render our results on the conservative side. Additionally, the variance in the volume of articles per disease category is noteworthy, a factor likely attributable to the scope of databases consulted. Predominantly focusing on English and Chinese publications may have led to the exclusion of pertinent research from other regions, underscoring the need for further studies to augment our conclusions. The methodologies for SPGs in the included studies acupuncture needle and electrode implantation differ significantly, yet both target SPGs. It was ensured that within each disease category, analysis was restricted to a single stimulation technique. Despite the broad variations in disease types and study parameters throughout the study, there was a maintained consistency within each disease classification, facilitating a focused examination of homogeneous groups, despite its inherent limitations.

The promise of SPGs extends well beyond its established efficacy in treating AR, IS, and CH, indicating its vast potential across a broader spectrum of diseases, particularly for conditions associated with head and face pain, as well as cerebrovascular diseases. In-depth mechanistic research is essential to unravel how SPGs achieves its therapeutic outcomes, including its influence on neural signal transmission, inflammation modulation, and the autonomic nervous system across various medical conditions. Concurrent technological advancements in SPGs methods, including devices and techniques, aim to refine treatment administration, elevate patient comfort, and enhance overall outcomes. This invites exploration into innovative stimulation patterns, intensities, and durations. Additionally, fostering multidisciplinary collaboration across neurology, immunology, pain management, and rehabilitation is crucial to gain a comprehensive perspective on SPGs' role in disease management and recovery. Focused research in these areas has the potential to significantly enhance our understanding and application of SPGs in clinical settings, providing renewed hope to individuals grappling with complex health challenges.

### 4.3 Conclusion

SPGs have shown potential in treating AR, IS, and CH, suggesting their utility in clinical settings might be beneficial. However, evidence remains preliminary, necessitating further, more comprehensive research to ascertain their effects across a range of conditions, including PTN, PCSO, RFP, and CTTH. While this analysis reveals the possible regulatory impact of SPGs on the nervous system, these insights should be viewed as preliminary, guiding future research rather than definitive evidence. Thus, while SPGs offer potential benefits, validating these findings through rigorous research is imperative.

## Data availability statement

The original contributions presented in the study are included in the article/Supplementary material, further inquiries can be directed to the corresponding authors.

## Author contributions

LQin: Data curation, Investigation, Methodology, Software, Writing—original draft, Writing—review & editing. DC: Data curation, Investigation, Validation, Writing—original draft. XL: Formal analysis, Methodology, Software, Supervision, Writing—review & editing, Data curation, Investigation, Validation. YG: Data curation, Formal analysis, Writing—review & editing. WX: Conceptualization, Writing—review & editing. HD: Conceptualization, Writing—review & editing. LQiu: Conceptualization, Writing—review & editing. JY: Funding acquisition, Project administration, Resources, Supervision, Writing—review & editing. LZ: Funding acquisition, Methodology, Project administration, Resources, Supervision, Validation, Writing—review & editing.
