# Supplementary material for: Sphenopalatine ganglion stimulation: a comprehensive evaluation across diseases in randomized controlled trials
Source: Front Neurol. 2024 May 15;15:1352145. doi: 10.3389/fneur.2024.1352145 (PMC11135047; doi:10.3389/fneur.2024.1352145)
Supplement: Supplementary file 1 [file Data_Sheet_1.pdf]

Supplementary Material

1 Supplementary Tables and Figures

1.1 Immunoglobulin E, IgE

IgE levels were assessed as an outcome in 4 articles. In terms of reducing IgE, the results of all three investigations showed that SPGs was not statistically significant when compared to WM. Further details are presented in Figure S1.

Study or Subgroup	SPGs Mean	SPGs SD	SPGs Total	WM Mean	WM SD	WM Total	Weight	Mean Difference IV, Random, 95% CI
HouF 2020	−111.93	40.21	30	−93.91	47.12	30	28.2%	−18.0200 [−40.1862, 4.1462]
LiYM 2007	−68.86	72.61	50	−39.64	81.21	50	18.2%	−29.2200 [−59.4152, 0.9752]
SongWJ 2020	−42.743	22.4	32	−39.421	24.49	32	53.6%	−3.3220 [−14.8212, 8.1772]
**Total (95% CI)**			**112**			**112**	**100.0%**	**−12.1721 [−26.7998, 2.4557]**

Heterogeneity: $Tau^2 = 69.45$; $Chi^2 = 3.29$, df = 2 (P = 0.19); $I^2 = 39\%$
Test for overall effect: Z = 1.63 (P = 0.10)

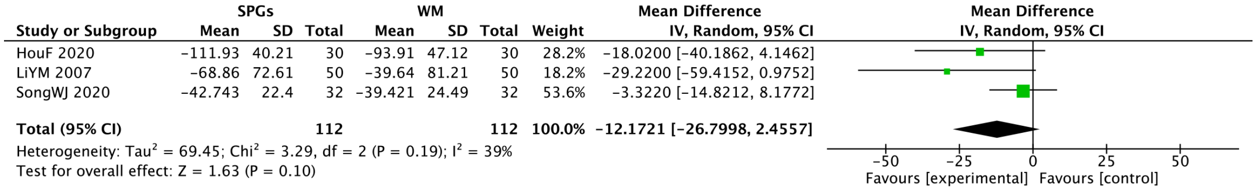

Fig.S 1: Forest plot for AR using SPGs: IgE as the outcome.

1.2 Cluster Headache, CH

Two RCTs were included in the assessment of SPGs for treating CH. In the studies reviewed, participants underwent implantation of the Autonomic Technologies Inc. Sphenopalatine Ganglion Neurostimulation System under local anesthesia. This device, comprising a miniaturized implant with a built-in lead containing six stimulating electrodes, was positioned adjacent to the SPG within the PPF. The primary outcome measure focused on the number of participants experiencing relief within 15 minutes of starting stimulation, while the secondary outcome measures the number of individuals whose pain disappeared within the same timeframe. The data reveal that SPGs treatment led to statistically significant improvements in both headache relief and disappearance within 15 minutes of starting the treatment, as evidenced by different levels of effectiveness across the studies. Despite high heterogeneity ($I^2 \geq 95\%$) indicating variation between study outcomes, individual studies like Goadsby PJ 2019 and Schoenen J 2013 demonstrate significant effects, with overall analyses yielding a statistically significant positive impact (P = 0.002) on CH symptoms. Hence, the evidence suggests that SPGs treatment may have a beneficial effect on alleviating and resolving headaches, warranting careful consideration of its application due to study variability. Further research with standardized methodologies is required to confirm these findings. Detailed results are presented in Figure S2.

1.3 Primary Trigeminal Neuralgia, PTN

Three RCTs were included to evaluate the effectiveness of SPGs in treating PTN. The primary outcome measures included improvements in headache intensity, assessed using the Visual Analog Scale (VAS), and an overall evaluation of effectiveness.

The comprehensive data analysis shows that SPGs have statistically significant improvements in headache

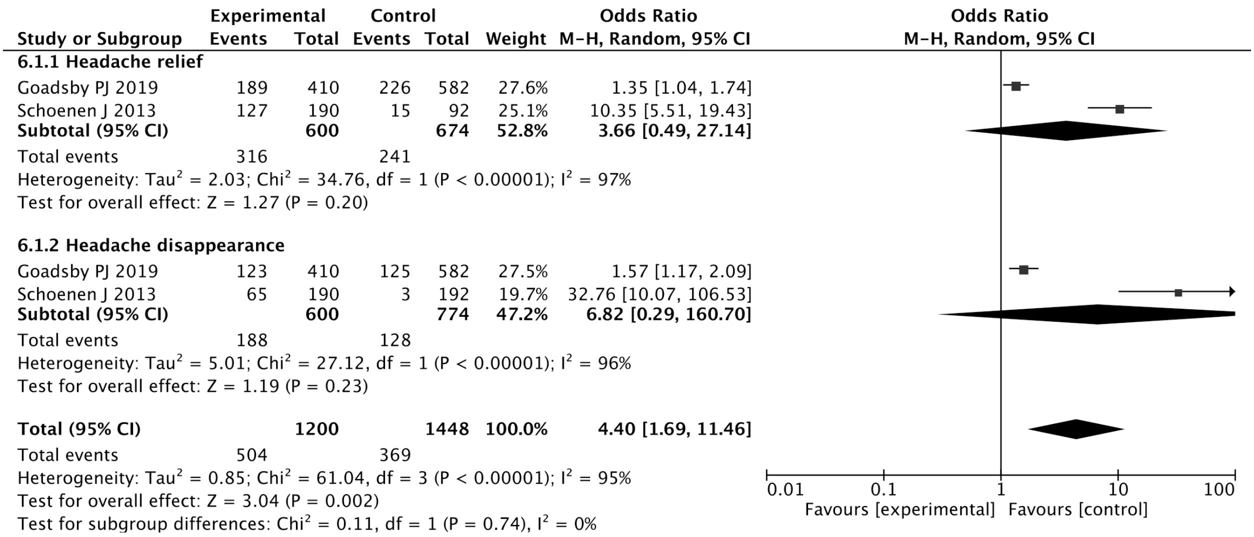

Fig.S 2: Forest Plot Showing Improvement in CH with SPGs.

intensity ($p<0.05$), with an overall mean difference of -0.97 (95% CI: [-1.51, -0.42]), indicating that the experimental group has significantly improved compared to the control group. Moreover, heterogeneity is very low ($I^2 = 0\%$), indicating consistent results across studies. Detailed results are available in Figure S3a. Regarding the efficacy rate improvement between SPGs and CA, the overall odds ratio is 2.18 (95% CI: [0.78, 6.08]), showing no statistically significant difference, indicating that there is no significant difference in the event occurrence rate between the experimental and control groups. Heterogeneity is also very low ($I^2 = 0\%$), indicating that the study results are consistent. Detailed results are available in Figure S3b.

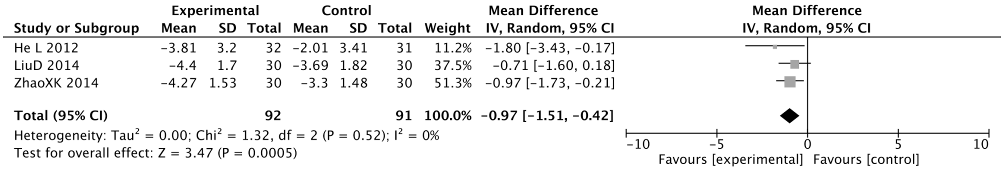

Fig.S 3a: Forest Plot Showing Headache Intensity Improvement in PTN with SPGs.

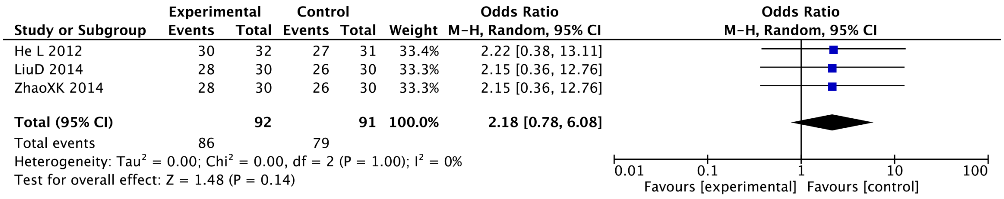

Fig.S 3b: Forest Plot of Efficacy Assessment in PTN with SPGs.

1.4 Normal nasal cavity function, NNCF

Three RCTs were conducted to assess the effect of SPGs on NNCF. Outcome measures were evaluated at three specific time points: 30 minutes, 2 hours, and 24 hours after stimulation. In these studies across three different time points, the experimental group showed significant improvements over the control group in reducing NAR, increasing NCV, modulating eNO, and elevating the levels of SP, VIP, and NPY. The results indicate that the experimental treatment had significant short-term and long-term effects on these

indicators ($P<0.05$). Additionally, the heterogeneity among all indicators at each time point was generally low, suggesting consistency in the research results. This demonstrates that the experimental intervention has a consistent effect on these biomarkers, both in the short term and extending up to 24 hours later. Detailed results are available in Figure 4a, Figure 4b and Figure 4c.

Table.S 1: Introduction to traditional Chinese medicine treatment methods.

SPGs	Stimulating the sphenopalatine ganglion using acupuncture needles: This involves the insertion of an acupuncture needle approximately 60mm in length into the Pterygopalatine Fossa. The direction of insertion is forward, upward, and inward, with a depth of about 55mm. When the needle tip approaches the vicinity of the sphenopalatine ganglion, a radiating sensation may be felt locally.
BSPGs	This is similar to SPGs but involves needling the sphenopalatine ganglion on both sides.
CA	This traditional method involves inserting acupuncture needles at specific acupoints. Commonly used acupoints for treating Allergic Rhinitis include Yingxiang, Yintang, Shangxing, and Hegu.
TCM	This includes the use of traditional Chinese herbal formulas based on the patient's symptoms and diagnostic patterns. Common formulas used in the treatment of Allergic Rhinitis include Longdan Xiegan Tang, Canger Zi San, and Yupingfeng San.

Table.S 2: Intervention Method and Stimulation Device Summary

Category	Study	Intervention Method	Stimulation Device	Usage Parameters
AR	ChenLQ 2015	Acupuncture at the sphenopalatine ganglion	0.35mm*60mm acupuncture needle, Dongbang brand	None
AR	LiGY 2020	Acupuncture at the sphenopalatine ganglion	Not specified	None
AR	ShaM 2021	Acupuncture at the sphenopalatine ganglion	0.35mm*55mm special rhinitis needle	None
AR	Mi JP 2020	Acupuncture at the sphenopalatine ganglion	0.3mm*75mm acupuncture needle	None
AR	ZhangL 2020	Acupuncture at the sphenopalatine ganglion	0.35mm*60mm acupuncture needle, Beijing Zhongyan brand	None
AR	ChenXY 2013	Acupuncture at the sphenopalatine ganglion	0.3mm*75mm acupuncture needle	Continuous wave, frequency 10Hz, maximum tolerable current, treatment time 15min
AR	FanYY 2018	Acupuncture at the sphenopalatine ganglion	Not specified	None
AR	WangX 2018	Acupuncture at the sphenopalatine ganglion	0.35mm*55mm acupuncture needle	None
AR	FuYN 2019	Acupuncture at the sphenopalatine ganglion	0.35mm*60mm acupuncture needle	None
AR	XuZX 2016	Acupuncture at the sphenopalatine ganglion	0.35mm*60mm acupuncture needle	None
AR	ZhangL 2015	Acupuncture at the sphenopalatine ganglion	0.35mm*60mm acupuncture needle, Beijing Zhongyan brand	None
AR	HouF 2020	Acupuncture at the sphenopalatine ganglion	0.35mm×55mm acupuncture needle, Diepalate brand	None
AR	LiYM 2007	Acupuncture at the sphenopalatine ganglion	Hua Tuo brand stainless steel No. 30 needle	Sparse-dense wave, frequency 80-100Hz, stimulation as tolerated by the patient.
AR	KanZY 2018	Acupuncture at the sphenopalatine ganglion	No. 7 puncture needle (10cm long)	None
AR	FengXX 2018	Acupuncture at the sphenopalatine ganglion	0.35mm×60.00mm acupuncture needle	None
AR	HuXL 2017	Acupuncture at the sphenopalatine ganglion	0.4mm×60mm acupuncture needle, Beijing Zhongyan	None
AR	LiKL 2018	Acupuncture at the sphenopalatine ganglion	0.35mm*60mm acupuncture needle, Hua Tuo brand	None
AR	TanLY 2020	Acupuncture at the sphenopalatine ganglion	0.35mm×60mm acupuncture needle, Hua Tuo brand	None
AR	SongWJ 2020	Acupuncture at the sphenopalatine ganglion	0.35mm×60mm acupuncture needle	None
AR	DongNX 2021	Acupuncture at the sphenopalatine ganglion	Not specified	None
AR	ShenL 2021	Acupuncture at the sphenopalatine ganglion	0.35mm×60mm acupuncture needle, Hua Tuo brand	None
IS	Bornstein NM 2019b	Implantation of neurostimulator electrodes	Neurostimulator electrodes (23mm long, 2mm diameter)	Not specified
IS	Bornstein NM 2019a	Implantation of neurostimulator electrodes	Neurostimulator electrodes (23mm long, 2mm diameter)	Not specified

Table 2 continued from previous page

Category	Study	Intervention Method	Stimulation Device	Usage Parameters
CH	Goadsby PJ 2019	Implantation of neurostimulator electrodes	Autonomic Technologies Inc. (ATI) Neurostimulator	Adjust electrical stimulation parameters every two weeks based on patient's numbness
CH	Schoenen J 2013	Implantation of neurostimulator electrodes	Autonomic Technologies Inc. (ATI) Neurostimulator	Three stimulation doses were used during the treatment adjustment period.
PTN	ZhaoXK 2014	Acupuncture at the sphenopalatine ganglion	0.35mm*65mm acupuncture needle, Hua Tuo brand	None
PTN	LiuD 2014	Acupuncture at the sphenopalatine ganglion	0.35mm × 65mm acupuncture needle	None
PTN	He L 2012	Acupuncture at the sphenopalatine ganglion	Not specified	Appropriate intensity for 30min
NNCF	WangKKJ 2016	Acupuncture at the sphenopalatine ganglion	0.35mm*60mm acupuncture needle, Dongbang Medical	None
NNCF	WangKJ 2016	Acupuncture at the sphenopalatine ganglion	0.35mm*60mm acupuncture needle, Dongbang Medical	None
NNCF	WangK 2015	Acupuncture at the sphenopalatine ganglion	0.35mm*60mm acupuncture needle, Dongbang Medical	None
PCSO	ChenLQ 2016	Acupuncture at the sphenopalatine ganglion	0.25mm*50mm acupuncture needle, Dongbang Medical	None
RFP	LuoL 2020	Acupuncture at the sphenopalatine ganglion	0.35mm×60mm acupuncture needle	None
CTTH	WangGJ 2020	Acupuncture at the sphenopalatine ganglion	0.35mm × 60mm acupuncture needle, Zhongyan brand	None
LF SPGs in CCH	Barloese M 2018	Implantation of neurostimulator electrodes	PulsanteTM, Autonomic Technologies Inc.	20Hz, usual intensity, duration 30min
LF SPGs in CCH	Guo S 2018	Implantation of neurostimulator electrodes	PulsanteTM SPG Microstimulator System	20Hz, 0.2-0.21mA, duration 30min

Table.S 3: Comparison between Neurostimulator Electrode Implantation and Acupuncture at the Sphenopalatine Ganglion

**Feature**	**Implantation of Neurostimulator Electrodes**	**Acupuncture at the Sphenopalatine Ganglion**
Definition	A small electrical stimulator device is surgically implanted near the sphenopalatine ganglion, where electrical pulses are transmitted through electrodes to stimulate the ganglion.	Specific length and diameter needles are used to physically stimulate the sphenopalatine ganglion directly, near the sphenopalatine fossa.
Method of Stimulation	Electrical stimulation allows for precise control over current intensity, frequency, and stimulation duration.	Physical stimulation relies on the position of the needle and the skill and experience of the practitioner.
Invasiveness	High, requiring surgical implantation and potential surgical removal.	Low, representing a non-invasive or minimally invasive treatment method.
Repeatability	The implanted device allows for long-term and repeated stimulation.	Each treatment session requires reinsertion of the needle, with repeatability depending on the practitioner's consistency and accuracy.
Adjustability	Treatment effects can be optimized by adjusting the device's parameters.	Lower adjustability, mainly dependent on technique and experience.
Potential Risks	Surgical risks, discomfort, or complications associated with the implant.	Lower risk of local discomfort, infection, or bleeding from needle insertion.
Treatment Objectives	Typically used for long-term management or treatment of intractable conditions, such as chronic headaches.	Widely applied for various conditions, including both acute and chronic pain management.
Cost and Accessibility	Higher cost, requiring professional medical equipment and surgical procedures.	Lower cost, simple operation, and feasible in various medical settings.

NAR

Study or Subgroup	Experimental Mean	Experimental SD	Experimental Total	Control Mean	Control SD	Control Total	Weight	Mean Difference IV, Random, 95% CI
WangK 2015	0.23	0.01	20	0.33	0.02	19	50.5%	−0.10 [−0.11, −0.09]
WangKJ 2016	0.2278	0.0125	25	0.3395	0.023	25	49.5%	−0.11 [−0.12, −0.10]
**Total (95% CI)**			**45**			**44**	**100.0%**	**−0.11 [−0.12, −0.09]**

Heterogeneity: $Tau^2 = 0.00$; $Chi^2 = 2.56$, $df = 1$ ($P = 0.11$); $I^2 = 61\%$
Test for overall effect: $Z = 18.08$ ($P < 0.00001$)

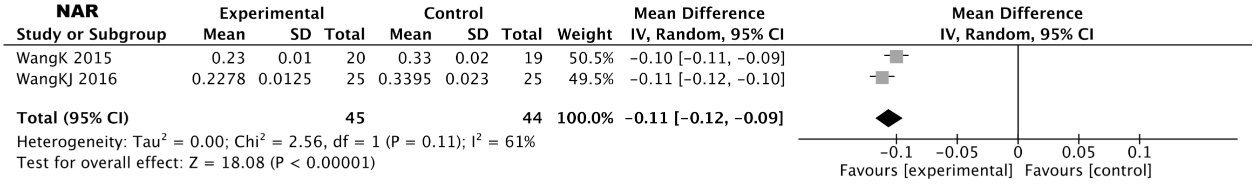

NCV

Study or Subgroup	Experimental Mean	Experimental SD	Experimental Total	Control Mean	Control SD	Control Total	Weight	Mean Difference IV, Random, 95% CI
WangK 2015	16.66	0.45	20	14.81	0.26	19	48.4%	1.85 [1.62, 2.08]
WangKJ 2016	16.66	0.455	25	15.15	0.21	25	51.6%	1.51 [1.31, 1.71]
**Total (95% CI)**			**45**			**44**	**100.0%**	**1.67 [1.34, 2.01]**

Heterogeneity: $Tau^2 = 0.05$; $Chi^2 = 4.87$, $df = 1$ ($P = 0.03$); $I^2 = 79\%$
Test for overall effect: $Z = 9.86$ ($P < 0.00001$)

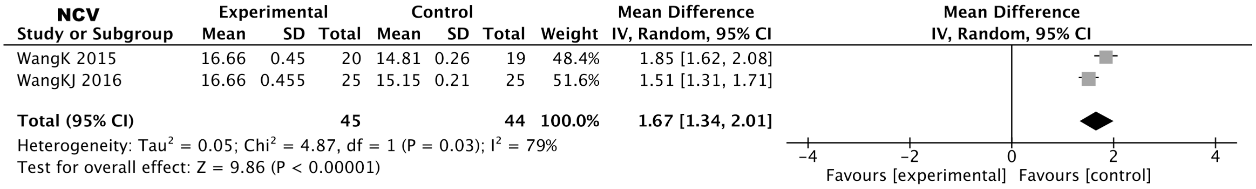

eNO

Study or Subgroup	Experimental Mean	Experimental SD	Experimental Total	Control Mean	Control SD	Control Total	Weight	Mean Difference IV, Random, 95% CI
WangK 2015	290.1	32.91	20	272.5	21.92	19	39.3%	17.60 [0.13, 35.07]
WangKJ 2016	292.2	28.9	25	269.9	21.22	25	60.7%	22.30 [8.25, 36.35]
**Total (95% CI)**			**45**			**44**	**100.0%**	**20.45 [9.50, 31.40]**

Heterogeneity: $Tau^2 = 0.00$; $Chi^2 = 0.17$, $df = 1$ ($P = 0.68$); $I^2 = 0\%$
Test for overall effect: $Z = 3.66$ ($P = 0.0003$)

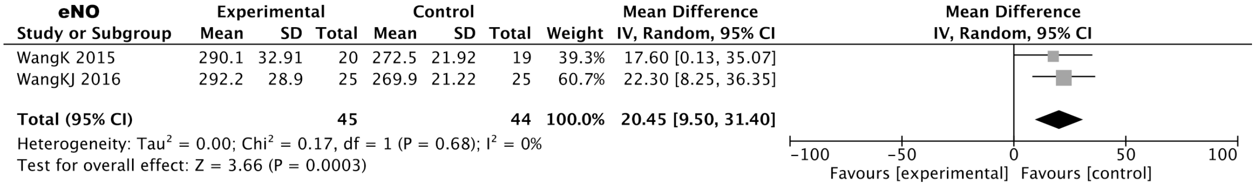

SP

Study or Subgroup	Experimental Mean	Experimental SD	Experimental Total	Control Mean	Control SD	Control Total	Weight	Mean Difference IV, Random, 95% CI
WangK 2015	511.5	68.24	20	345.1	29.89	19	44.4%	166.40 [133.61, 199.19]
WangKKJ 2016	509.8	67.88	25	339.5	31.4	25	55.6%	170.30 [140.98, 199.62]
**Total (95% CI)**			**45**			**44**	**100.0%**	**168.57 [146.71, 190.42]**

Heterogeneity: $Tau^2 = 0.00$; $Chi^2 = 0.03$, $df = 1$ ($P = 0.86$); $I^2 = 0\%$
Test for overall effect: $Z = 15.12$ ($P < 0.00001$)

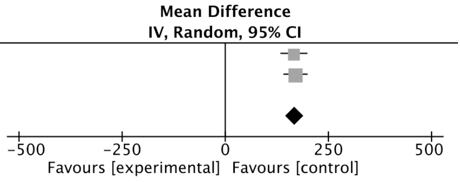

VIP

Study or Subgroup	Experimental Mean	Experimental SD	Experimental Total	Control Mean	Control SD	Control Total	Weight	Mean Difference IV, Random, 95% CI
WangK 2015	0.47	0.05	20	0.42	0.05	19	47.7%	0.05 [0.02, 0.08]
WangKKJ 2016	0.4952	0.0525	25	0.4185	0.0533	25	52.3%	0.08 [0.05, 0.11]
**Total (95% CI)**			**45**			**44**	**100.0%**	**0.06 [0.04, 0.09]**

Heterogeneity: $Tau^2 = 0.00$; $Chi^2 = 1.48$, $df = 1$ ($P = 0.22$); $I^2 = 33\%$
Test for overall effect: $Z = 4.80$ ($P < 0.00001$)

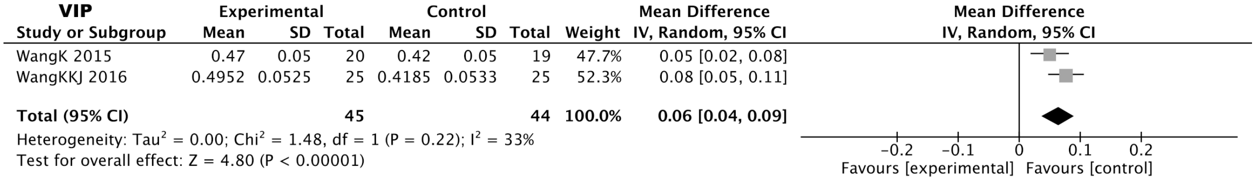

NPY

Study or Subgroup	Experimental Mean	Experimental SD	Experimental Total	Control Mean	Control SD	Control Total	Weight	Mean Difference IV, Random, 95% CI
WangK 2015	12.94	2.56	20	6.64	1.02	19	42.0%	6.30 [5.09, 7.51]
WangKKJ 2016	12.76	2.484	25	6.204	0.8746	25	58.0%	6.56 [5.52, 7.59]
**Total (95% CI)**			**45**			**44**	**100.0%**	**6.45 [5.66, 7.23]**

Heterogeneity: $Tau^2 = 0.00$; $Chi^2 = 0.10$, $df = 1$ ($P = 0.75$); $I^2 = 0\%$
Test for overall effect: $Z = 16.08$ ($P < 0.00001$)

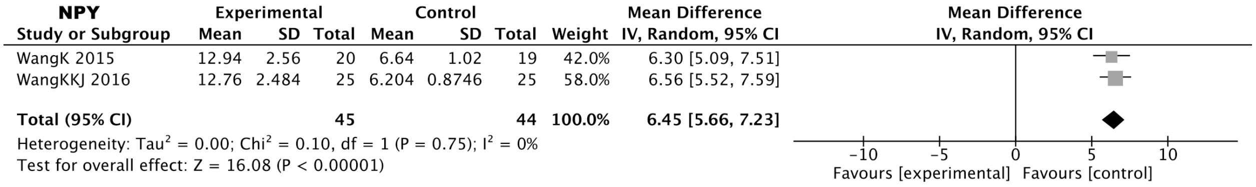

Fig.S 4a: Forest Plot of the Impact of SPGs on NNCF at 30 Minutes.

NAR

Study or Subgroup	Experimental Mean	Experimental SD	Experimental Total	Control Mean	Control SD	Control Total	Weight	Mean Difference IV, Random, 95% CI
WangK 2015	0.24	0.01	20	0.36	0.02	19	58.0%	−0.12 [−0.13, −0.11]
WangKJ 2016	0.2447	0.0142	25	0.3614	0.0264	25	42.0%	−0.12 [−0.13, −0.10]
**Total (95% CI)**			**45**			**44**	**100.0%**	**−0.12 [−0.13, −0.11]**

Heterogeneity: $Tau^2 = 0.00$; $Chi^2 = 0.18$, df = 1 (P = 0.68); $I^2 = 0\%$
Test for overall effect: Z = 30.52 (P < 0.00001)

Mean Difference IV, Random, 95% CI

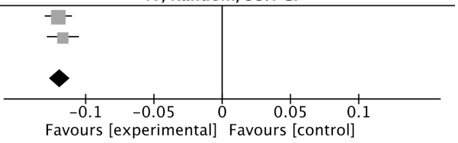

NCV

Study or Subgroup	Experimental Mean	Experimental SD	Experimental Total	Control Mean	Control SD	Control Total	Weight	Mean Difference IV, Random, 95% CI
WangK 2015	16.47	0.49	20	14.79	0.28	19	44.3%	1.68 [1.43, 1.93]
WangKJ 2016	16.47	0.4908	25	14.81	0.2825	25	55.7%	1.66 [1.44, 1.88]
**Total (95% CI)**			**45**			**44**	**100.0%**	**1.67 [1.50, 1.83]**

Heterogeneity: $Tau^2 = 0.00$; $Chi^2 = 0.01$, df = 1 (P = 0.91); $I^2 = 0\%$
Test for overall effect: Z = 19.74 (P < 0.00001)

Mean Difference IV, Random, 95% CI

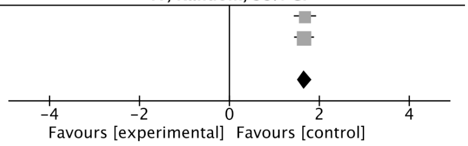

eNO

Study or Subgroup	Experimental Mean	Experimental SD	Experimental Total	Control Mean	Control SD	Control Total	Weight	Mean Difference IV, Random, 95% CI
WangK 2015	233.6	16.49	20	275	19	19	44.0%	−41.40 [−52.59, −30.21]
WangKJ 2016	233.6	16.26	25	277.1	19.39	25	56.0%	−43.50 [−53.42, −33.58]
**Total (95% CI)**			**45**			**44**	**100.0%**	**−42.58 [−50.00, −35.15]**

Heterogeneity: $Tau^2 = 0.00$; $Chi^2 = 0.08$, df = 1 (P = 0.78); $I^2 = 0\%$
Test for overall effect: Z = 11.24 (P < 0.00001)

Mean Difference IV, Random, 95% CI

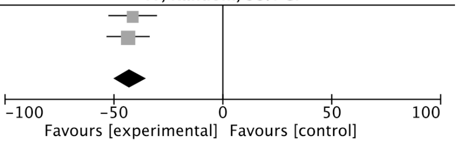

SP

Study or Subgroup	Experimental Mean	Experimental SD	Experimental Total	Control Mean	Control SD	Control Total	Weight	Mean Difference IV, Random, 95% CI
WangK 2015	410.8	83.86	20	357.1	27.46	19	42.3%	53.70 [14.93, 92.47]
WangKKJ 2016	419	80.26	25	354.6	26.86	25	57.7%	64.40 [31.22, 97.58]
**Total (95% CI)**			**45**			**44**	**100.0%**	**59.88 [34.67, 85.08]**

Heterogeneity: $Tau^2 = 0.00$; $Chi^2 = 0.17$, df = 1 (P = 0.68); $I^2 = 0\%$
Test for overall effect: Z = 4.66 (P < 0.00001)

Mean Difference IV, Random, 95% CI

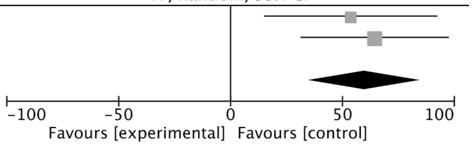

VIP

Study or Subgroup	Experimental Mean	Experimental SD	Experimental Total	Control Mean	Control SD	Control Total	Weight	Mean Difference IV, Random, 95% CI
WangK 2015	0.49	0.05	20	0.42	0.05	19	44.1%	0.07 [0.04, 0.10]
WangKKJ 2016	0.4807	0.0542	25	0.3998	0.046	25	55.9%	0.08 [0.05, 0.11]
**Total (95% CI)**			**45**			**44**	**100.0%**	**0.08 [0.06, 0.10]**

Heterogeneity: $Tau^2 = 0.00$; $Chi^2 = 0.26$, df = 1 (P = 0.61); $I^2 = 0\%$
Test for overall effect: Z = 7.16 (P < 0.00001)

Mean Difference IV, Random, 95% CI

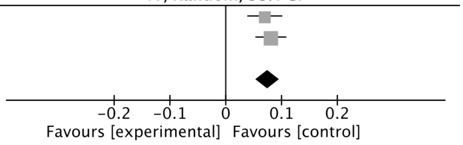

NPY

Study or Subgroup	Experimental Mean	Experimental SD	Experimental Total	Control Mean	Control SD	Control Total	Weight	Mean Difference IV, Random, 95% CI
WangK 2015	12.91	1.96	20	5.9	0.84	19	42.7%	7.01 [6.07, 7.95]
WangKKJ 2016	12.74	1.919	25	5.777	0.7721	25	57.3%	6.96 [6.15, 7.77]
**Total (95% CI)**			**45**			**44**	**100.0%**	**6.98 [6.37, 7.60]**

Heterogeneity: $Tau^2 = 0.00$; $Chi^2 = 0.01$, df = 1 (P = 0.94); $I^2 = 0\%$
Test for overall effect: Z = 22.31 (P < 0.00001)

Mean Difference IV, Random, 95% CI

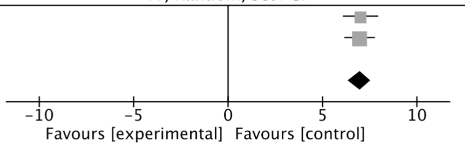

Fig.S 4b: Forest Plot of the Impact of SPGs on NNCF at 2 Hours.

NAR

Study or Subgroup	Experimental Mean	Experimental SD	Experimental Total	Control Mean	Control SD	Control Total	Weight	Mean Difference IV, Random, 95% CI
WangK 2015	0.28	0.02	20	0.35	0.02	19	49.2%	−0.07 [−0.08, −0.06]
WangKJ 2016	0.2824	0.0209	25	0.3533	0.0236	25	50.8%	−0.07 [−0.08, −0.06]
**Total (95% CI)**			**45**			**44**	**100.0%**	**−0.07 [−0.08, −0.06]**

Heterogeneity: Tau^2 = 0.00; Chi^2 = 0.01, df = 1 (P = 0.92); I^2 = 0%
Test for overall effect: Z = 15.68 (P < 0.00001)

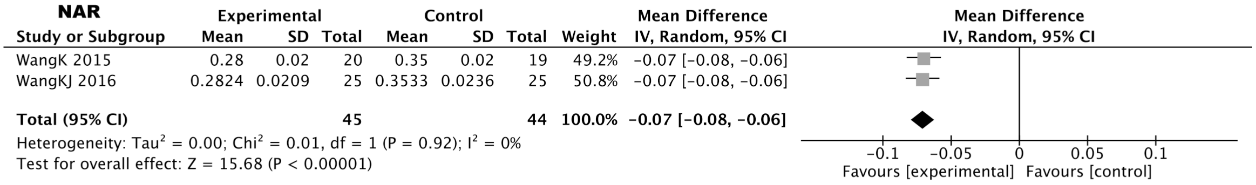

NCV

Study or Subgroup	Experimental Mean	Experimental SD	Experimental Total	Control Mean	Control SD	Control Total	Weight	Mean Difference IV, Random, 95% CI
WangK 2015	15.84	0.33	20	14.93	0.24	19	46.5%	0.91 [0.73, 1.09]
WangKJ 2016	15.84	0.3398	25	14.79	0.2494	25	53.5%	1.05 [0.88, 1.22]
**Total (95% CI)**			**45**			**44**	**100.0%**	**0.98 [0.85, 1.12]**

Heterogeneity: Tau^2 = 0.00; Chi^2 = 1.26, df = 1 (P = 0.26); I^2 = 20%
Test for overall effect: Z = 14.10 (P < 0.00001)

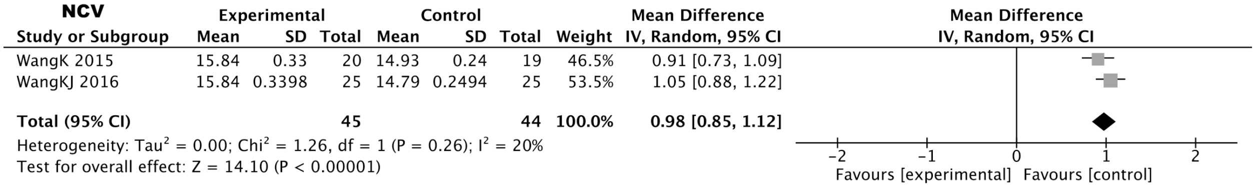

eNO

Study or Subgroup	Experimental Mean	Experimental SD	Experimental Total	Control Mean	Control SD	Control Total	Weight	Mean Difference IV, Random, 95% CI
WangK 2015	184	10.98	20	276.4	18.82	19	41.6%	−92.40 [−102.13, −82.67]
WangKJ 2016	188.6	10.6	25	274.9	18.1	25	58.4%	−86.30 [−94.52, −78.08]
**Total (95% CI)**			**45**			**44**	**100.0%**	**−88.84 [−95.12, −82.56]**

Heterogeneity: Tau^2 = 0.00; Chi^2 = 0.88, df = 1 (P = 0.35); I^2 = 0%
Test for overall effect: Z = 27.72 (P < 0.00001)

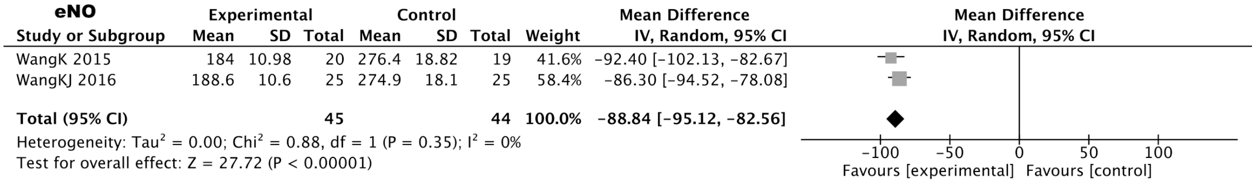

SP

Study or Subgroup	Experimental Mean	Experimental SD	Experimental Total	Control Mean	Control SD	Control Total	Weight	Mean Difference IV, Random, 95% CI
WangK 2015	375.9	35.23	20	343.2	24.26	19	43.0%	32.70 [13.80, 51.60]
WangKKJ 2016	385.4	34.18	25	340.7	24.22	25	57.0%	44.70 [28.28, 61.12]
**Total (95% CI)**			**45**			**44**	**100.0%**	**39.54 [27.14, 51.94]**

Heterogeneity: Tau^2 = 0.00; Chi^2 = 0.88, df = 1 (P = 0.35); I^2 = 0%
Test for overall effect: Z = 6.25 (P < 0.00001)

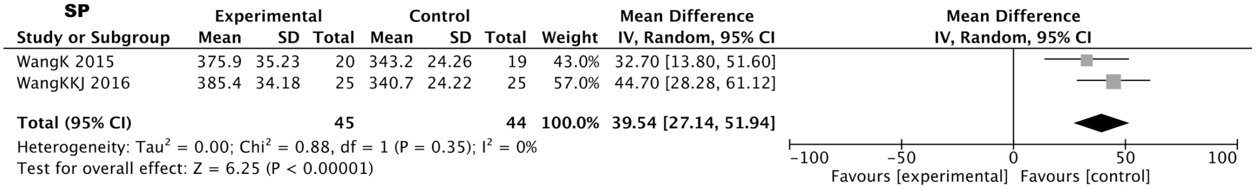

VIP

Study or Subgroup	Experimental Mean	Experimental SD	Experimental Total	Control Mean	Control SD	Control Total	Weight	Mean Difference IV, Random, 95% CI
WangK 2015	0.46	0.05	20	0.41	0.03	19	41.3%	0.05 [0.02, 0.08]
WangKKJ 2016	0.4376	0.0433	25	0.3944	0.0341	25	58.7%	0.04 [0.02, 0.06]
**Total (95% CI)**			**45**			**44**	**100.0%**	**0.05 [0.03, 0.06]**

Heterogeneity: Tau^2 = 0.00; Chi^2 = 0.16, df = 1 (P = 0.69); I^2 = 0%
Test for overall effect: Z = 5.45 (P < 0.00001)

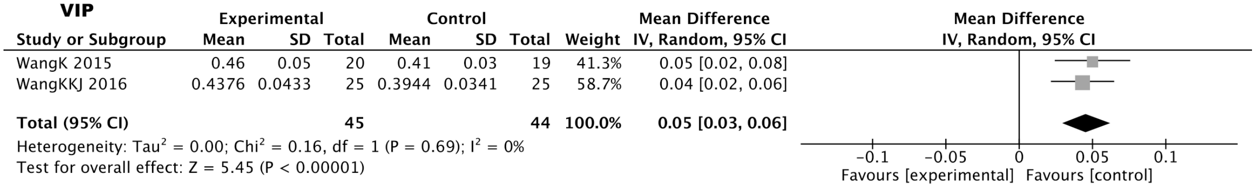

NPY

Study or Subgroup	Experimental Mean	Experimental SD	Experimental Total	Control Mean	Control SD	Control Total	Weight	Mean Difference IV, Random, 95% CI
WangK 2015	8.9	2.12	20	5.86	0.85	19	43.7%	3.04 [2.04, 4.04]
WangKKJ 2016	8.84	2.126	25	5.677	0.7635	25	56.3%	3.16 [2.28, 4.05]
**Total (95% CI)**			**45**			**44**	**100.0%**	**3.11 [2.44, 3.77]**

Heterogeneity: Tau^2 = 0.00; Chi^2 = 0.03, df = 1 (P = 0.86); I^2 = 0%
Test for overall effect: Z = 9.17 (P < 0.00001)

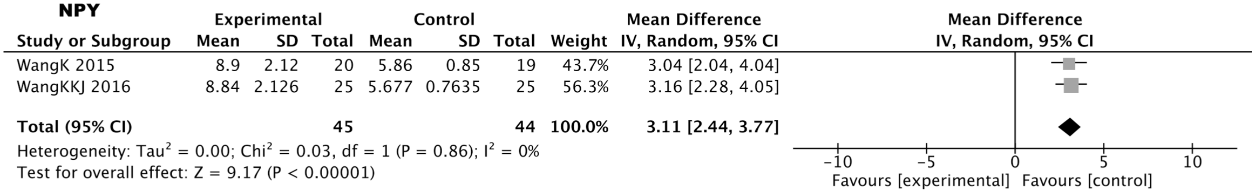

Fig.S 4c: Forest Plot of the Impact of SPGs on NNCF at 24 Hours.
